# Decoding Order and Disorder in Proteins by NMR Spectroscopy

**DOI:** 10.1021/jacs.4c14959

**Published:** 2025-04-14

**Authors:** Lorenzo Bracaglia, Silvia Oliveti, Isabella C. Felli, Roberta Pierattelli

**Affiliations:** Department of Chemistry “Ugo Schiff” and Magnetic Resonance Center, University of Florence, Sesto Fiorentino 50019, Italy

## Abstract

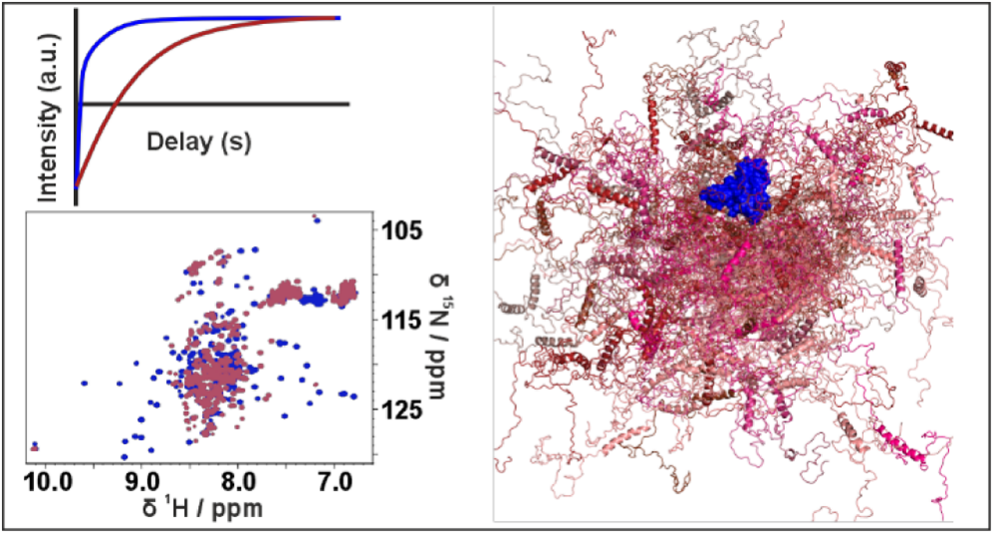

Proteins often have a complex architecture, consisting of both globular ordered domains and intrinsically disordered regions (IDRs). These multidomain proteins pose challenges for traditional structural biology techniques. One major difficulty arises from the dynamic and flexible nature of IDRs, which lack a stable three-dimensional structure. Indeed, this feature further complicates the application of traditional structural biology techniques. Characterizing these systems is typically simplified by isolating individual domains, which can provide valuable insights into the structure and function of specific regions. However, this approach overlooks the interactions and regulatory mechanisms that occur between domains. To capture the full functional and structural complexity of multidomain proteins, it is crucial to study larger constructs. In this study, we focused on the CREB binding protein (CBP), a pivotal protein involved in numerous cellular processes. CBP is characterized by its modular structure, featuring alternating globular domains and IDRs. We specifically examined the TAZ4 construct, encompassing the TAZ2 globular domain and the ID4 flexible linker region. To characterize this multidomain system, we designed NMR experiments that take advantage of the dynamic differences between the two domains to obtain 2D and 3D spectra enabling the selection of the signals based on their nuclear relaxation properties. These experiments allowed the sequence-specific assignment of the TAZ4 construct to be extended revealing a crosstalk between the disordered region and the globular domain.

## Introduction

**Figure 1:**
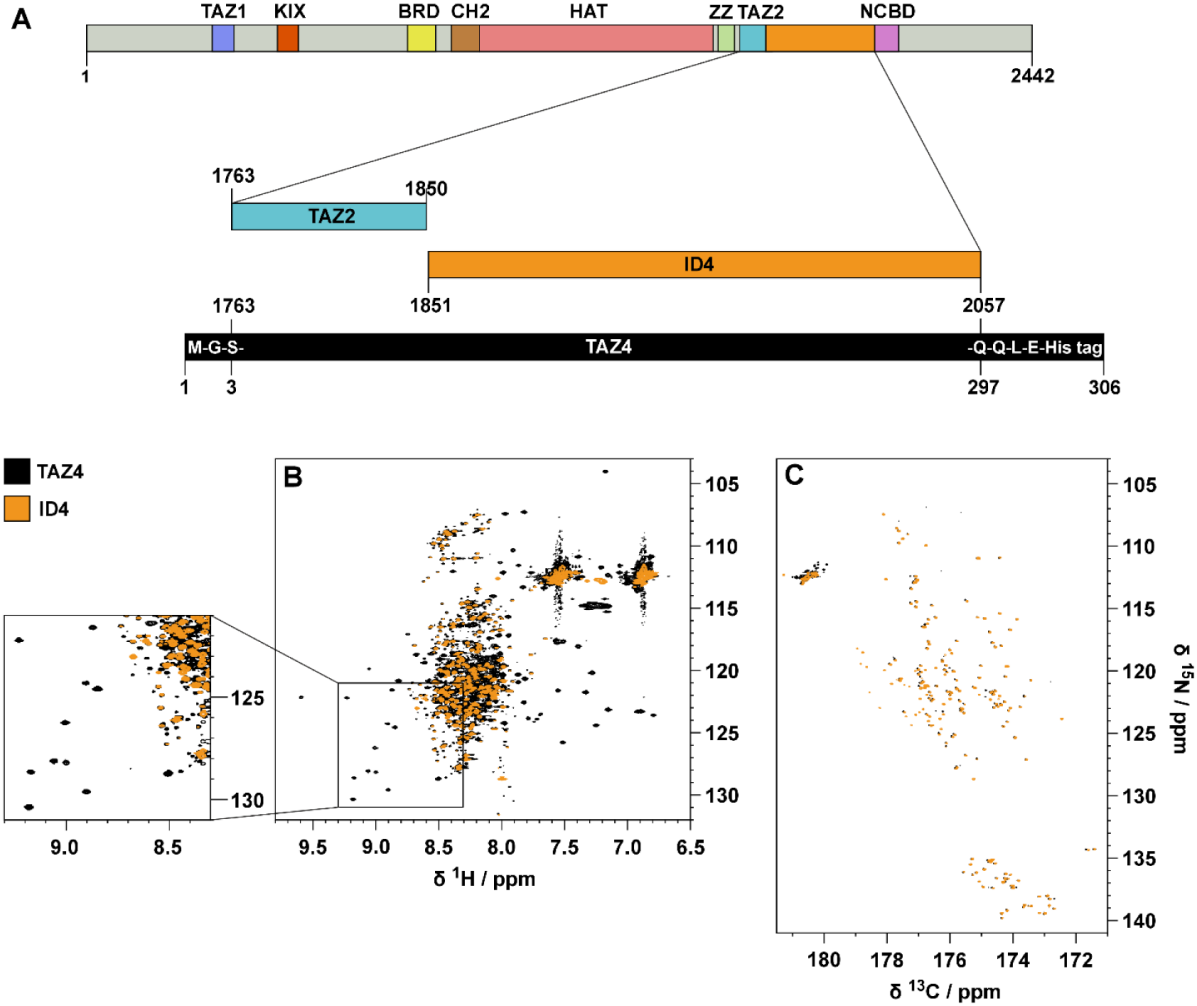
(A) Schematic representation of CBP domains distribution. The black bar on the bottom of the panel represents the construct discussed in this work (TAZ4). The numbers above the bar refer to the absolute sequence numbering of CBP, while those below the bar are relative to the construct itself. (B) ^1^H–^15^N HSQC spectra of TAZ4 (black) and ID4 (orange) recorded at 298 K and 16.4 T (700 MHz). The panel on the left is a zoom of a region of the HSQC. Due to the differences in NMR properties of the two domains, the contour levels needed to see the peaks of the TAZ2 domain in the TAZ4 construct are very low and very different from the optimal settings needed to observe resolved cross peaks for ID4 within the same construct. (C) ^13^C-^15^N CON spectra of TAZ4 (black) and ID4 (orange) recorded at 298 K and 16.4 T (700 MHz).

CBP and its paralog E1A-binding protein (p300) are modular transcriptional coactivators that increase gene expression by binding to transcription factors that contain DNA binding domains and, in turn, can control the rate of transcription of genetic information. CBP activity is found in almost all known cellular functions, such as the decision to grow, differentiate, or lead to cell death by apoptosis;^1−3^ in order to fulfill the variety of these processes, consistent with their complexity, many intracellular factors, including transcription factors, nuclear receptors, and other coactivators, have been found to interact with CBP. The domain architecture of its 2,442 residues is reported in Figure 1A. There are seven domains that are able to fold independently, and four of them require zinc binding to stabilize their tertiary structures: the transcriptional-adaptor zinc-finger-1 (TAZ1) domain,^4^ the plant homeodomain (PHD),^5^ a zinc-binding domain near the dystrophin WW domain (ZZ),^6^ and the transcriptional-adaptor zinc-finger-2 (TAZ2) domain;^7^ the other folded domains are KID-binding domain (KIX),^8^ the bromodomain,^9^ and the histone acetyltransferase domain (HAT).^10^ The nuclear-receptor coactivator-binding domain (NCBD) is intrinsically disordered but undergoes synergistic folding on forming a complex with its partners.^11^ Furthermore, there are five disordered regions of different length, denoted ID (which stands for “Intrinsically Disordered”) followed by the sequential number of the disordered region in the primary structure, which account for about 60% of the sequence. The ID regions (IDRs) have always been defined just as flexible linkers of proteins that connect functional regions (globular domains), providing their appropriate spatial separation. They are now emerging as possible functional motifs themselves involved in binding with partners.^12,13^ In light of the increasing interest in the investigation of intrinsically disordered proteins, in recent years three IDRs of CBP, namely IDR3,^12^ IDR4^14^ and IDR5,^15^ have been investigated by NMR spectroscopy to determine their structural and dynamic features. Moreover, their interactions with potential partners/interactors have been characterized by NMR and other ancillary techniques such as X-ray scattering and mass spectrometry, demonstrating that the IDRs provide additional opportunities for CBP to orchestrate its function.^12,13,15^ With a high incidence of Pro (16%), Gln (15%), Ser (11%), Gly (9%), and Ala (9%) residues, these IDRs clearly exhibit the compositional bias typical of disordered regions.^16−19^ ID4, the 207-residue long linker placed between the TAZ2 domain and the NCBD domain, is the CBP region with the highest percentage of Pro residues (22%); its biased sequence properties, together with other peculiar characteristics of IDRs contributed to render the sequence-specific assignment and the structural and dynamic characterization of ID4 quite challenging. This was recently achieved combining ^1^H and ^13^C direct detected NMR experiments,^14^ and the results showed that ID4 is highly flexible except for two regions encompassing residues 1852–1876 and 1952–1979, which have a high propensity to sample an α-helical conformation. The first helical region in the disordered fragment can be seen as the extension in the wild-type protein of the TAZ2 domain, whose structure has a peculiar fold composed of four alpha helices (α_1_, α_2_, α_3_, and α_4_) that are arranged in a helical bundle.^7^ The two partially populated helices of ID4 will thus be referred to as α_5_ and α_6_. Three zinc ions are coordinated by the TAZ2 domain, each bound to one histidine and three cysteine ligands in HCCC-type motifs.^7^ The zinc ions play a crucial role in stabilizing the TAZ2 helical bundle and maintaining its structure. They also contribute to the formation of the TAZ2 binding surface, which is essential for protein–protein interactions. We would like now to extend our understanding of the interplay between TAZ2 and ID4 by studying a novel construct comprising both regions, counting 295 amino acids (TAZ4). To this end novel NMR tools are presented to address the spectral complexity deriving from the simultaneous presence of ordered and disordered domains in the protein construct of interest.

## Results

The two domains of this construct exhibit distinct structural and dynamic features that are reflected on the NMR observables. The 2D HN spectrum of TAZ4 reported in Figure 1B shows two sets of signals: narrow and intense peaks with ^1^H resonance frequencies between 7.8 and 8.7 ppm (deriving from the ID4 region), and broader peaks that fall in a much wider range of chemical shifts (deriving from the TAZ2 domain). The spectrum is very crowded and many peaks of TAZ2 are hidden by the more intense ID4 peaks.

The resonances of the amino acids in the disordered region can be selectively observed with a ^13^C-detected 2D CON experiment, such as the one reported in Figure 1C. Indeed, the long coherence transfer delays (tuned to 1/2^1^J_CN_), intrinsic to the experiment, act as a transverse relaxation filter: signals of the residues in the globular domain are characterized by much faster transverse relaxation with respect to those of the highly flexible disordered regions and thus their intensity is greatly reduced, up to complete disappearance of the cross peaks. CON spectra thus reveal in a very clean way signals of the intrinsically disordered regions also when part of more complex protein constructs.^20^ Furthermore, the cross peaks in the spectrum are well resolved thanks to the narrow line widths (especially those of ^15^N) and to the long acquisition and evolution times used to build the direct and indirect dimensions, respectively.

In contrast, the selection of the signals of the globular domain, that should be accomplished through the 2D HN spectrum, is more challenging, particularly in the spectral region where signals from the highly flexible disordered region are clustered. However, it is possible to enhance the readability of the spectrum by taking advantage of the different relaxation properties of the nuclei of the two domains (TAZ2 and ID4). The implementation of transverse relaxation filters would select signals of the highly flexible region at the expense of those of the globular domain. Conversely, it is necessary to design a novel approach that enables the opposite selection to discriminate the signals of residues belonging to the more ordered regions with respect to the signals of the disordered ones. To this end we focused on ^1^H longitudinal relaxation aiming to exploit one of the properties that take advantage of the increased local correlation time typical of globular ordered domains: longitudinal relaxation enhancement upon selective perturbation of a subset of signals (amide protons in the present case).

### A Relaxation Filter Based on Longitudinal Relaxation Enhancement (LRE)

**Figure 2:**
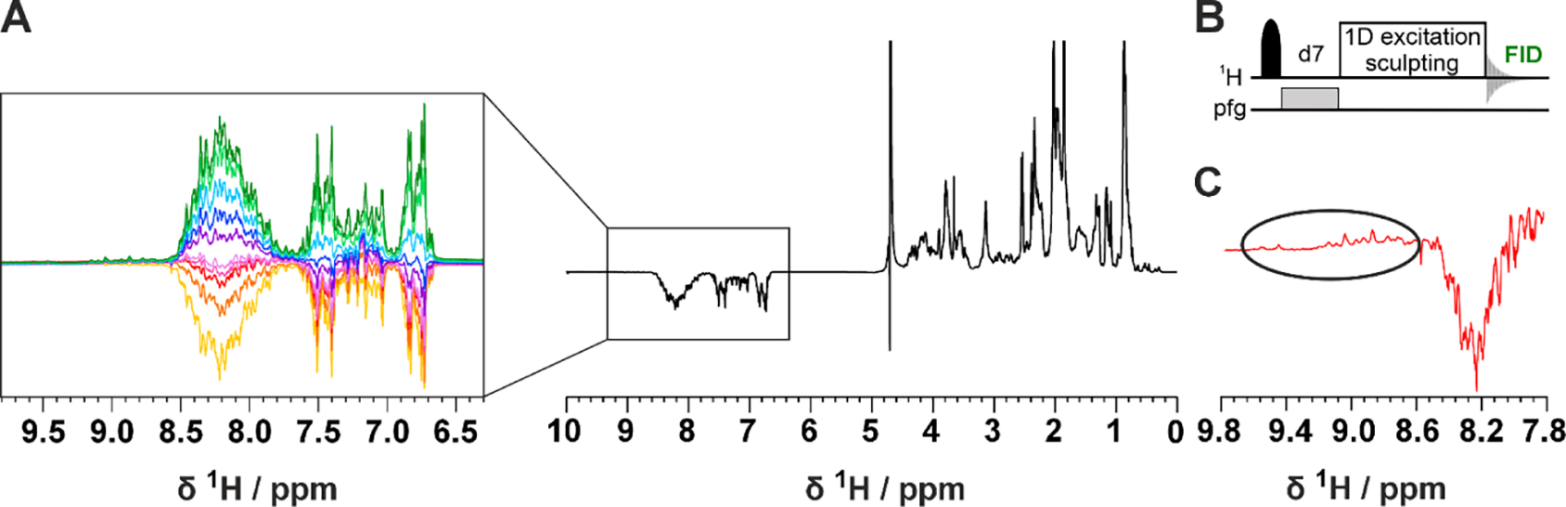
(A) The black spectrum on the right part of the panel is the inversion recovery ^1^H 1D spectrum of TAZ4 recorded at 298 K and 21.1 T (900 MHz) acquired with a band-selective ^1^H^N^ inversion pulse and with a recovery delay (d7) of 20 ms. The box on the leftmost part of the panel shows the same experiment acquired with different values of recovery delay: 20 ms (yellow), 100 ms (orange), 150 ms (red), 180 ms (hot pink), 200 ms (pink), 300 ms (violet), 400 ms (blue), 600 ms (cyan), 1.2 s (light green), 3.0 s (dark green). (B) Scheme of the pulse sequence used to record the spectra in panel A. The detailed pulse sequence is described in Figure S1. The black shape on the ^1^H channel is a band-selective π pulse on amide protons, followed by a variable recovery delay. The increase of the variable delay leads to the recovery of the magnetization. (C) The inversion recovery ^1^H 1D spectrum of TAZ4 recorded with a recovery delay of 150 ms is shown. The positive signals included in the highlighted spectral region belong to the TAZ2 domain, while the negative ones belong (mainly) to the ID4 domain. This shows that it is possible to selectively invert the sign of the peaks that originate from the disordered domain.

**Figure 3:**
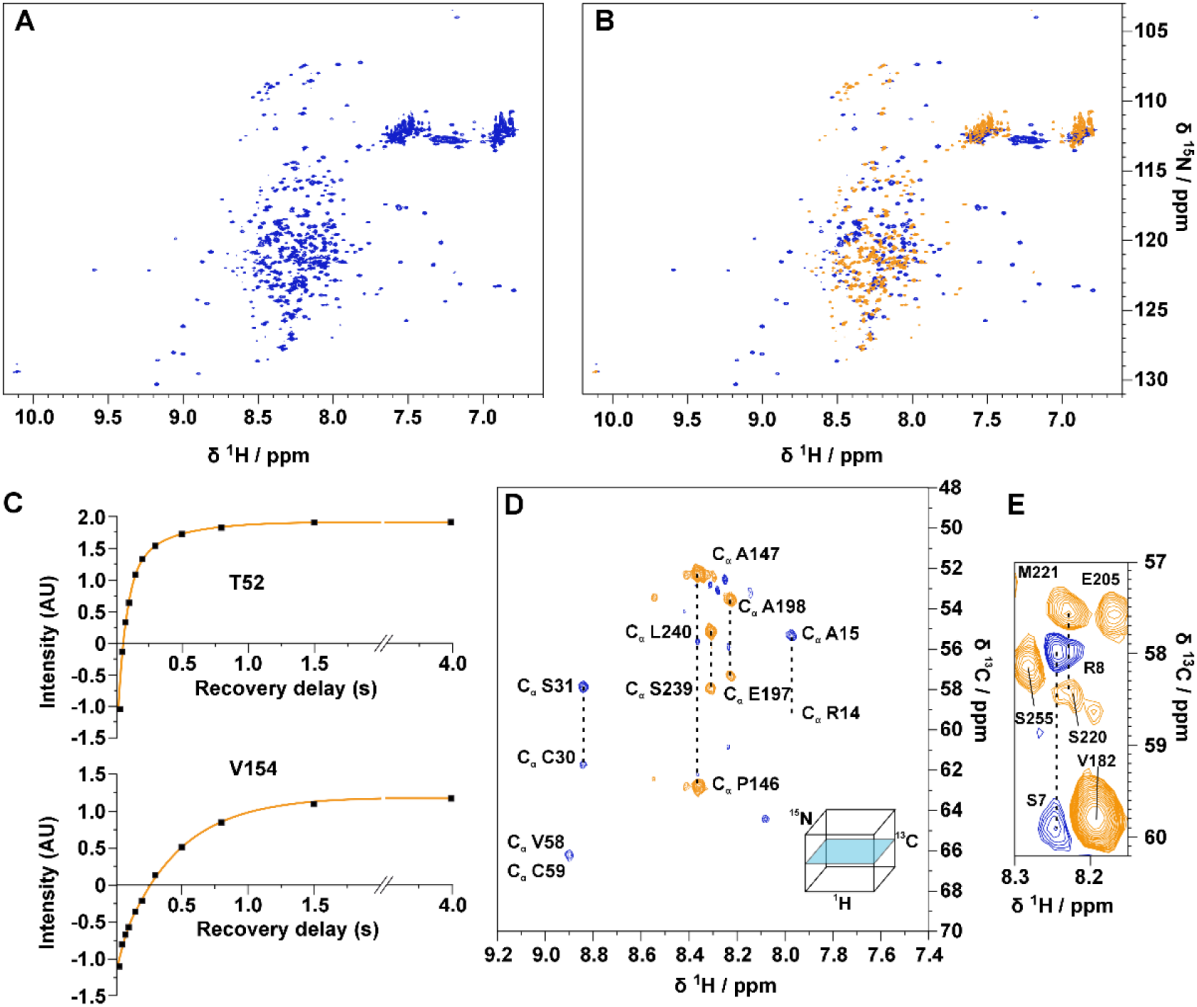
Inversion recovery ^1^H–^15^N HSQC spectrum (2D IR-HN) recorded at 298 K and 28.2 T (1200 MHz) with (A) a recovery delay of 4 s and (B) a recovery delay of 150 ms. Positive cross peaks are depicted with blue contours, while negative cross peaks are shown with orange contours. The pulse sequence is described in Figure S2. (C) The profile on top belongs to residue T52 (globular domain) and it shows a faster recovery than the one on the bottom which belongs to residue V154 (disordered region). (D) Inversion recovery 3D HNCA (3D IR-HNCA) spectrum recorded at 298 K and 21.1 T (900 MHz). Each signal displayed in the ^1^H^N^-^13^C^α^ plane extracted at 123.4 ppm in the ^15^N dimension from the 3D spectrum (sketched on the bottom right angle of the spectrum), is labeled with its assignment. (E) Detail taken from the 122.8 ppm ^15^N plane of the 3D IR-HNCA spectrum, showing an example of cross peaks deriving from two distinct regions of TAZ4 which can be distinguished by their difference in sign. The dotted vertical lines link the signals that share the same ^1^H^N^ resonance (positive cross peaks are depicted with blue contours, while negative cross peaks are shown with orange contours).

Figure 2A shows a series of 1D experiments in which a band selective inversion recovery block on amide protons has been implemented at the beginning of the pulse sequence (Figures 2B and S1). Perturbation of a selected subset of nuclear spins promotes longitudinal relaxation enhancement (LRE) in proteins, which increases with increasing local correlation time under conditions of low solvent exchange.^21^ Fine tuning of the recovery delay (d7) enables the discrimination of the TAZ2 ^1^H signals, which recover faster and result in positive signals, from the ID4 signals that exhibit a slower recovery and are still negative as shown in Figure 2C. This relaxation filter can be implemented in all the pulse sequences needed for the characterization of TAZ4. In Figure 3A,B, the 2D ^1^H–^15^N HSQC inversion recovery spectra (2D IR-HN) acquired with different recovery delays are shown. The pulse sequence is described in detail in Figure S2. This experiment was used to acquire residue-resolved inversion recovery profiles for the amide protons under band selective inversion. As an example, two inversion recovery profiles of representative residues in the TAZ2 and ID4 regions of the construct (T52 and V154) are shown in Figure 3C (the results obtained in this way for the whole TAZ4 construct are shown in Figure S3). A very different behavior is observed for amide protons in the globular domain (fast longitudinal recovery), with respect to those of the disordered region (slow longitudinal recovery in the experimental conditions used in the present work in which the contribution from the solvent exchange is modest). This property is exploited to encode a difference in sign of the observed cross peaks by appropriate selection of the recovery delay. In the spectrum acquired with the recovery delay of 150 ms the signals deriving from the TAZ2 domain are positive, while the vast majority of those deriving from the ID4 domain are negative. Some fast-relaxing peaks that were difficult to observe in the conventional 2D HN spectrum are now clearly visible (Figure S4). The implementation of the IR filter results in a reduction in intrinsic experimental sensitivity. In general the selection of the most appropriate delay is guided by maximizing the intensities of the cross peaks of residues in the globular domain; the cross peaks originating from residues in disordered regions are generally very intense, therefore even pronounced reductions in intensity caused by the IR filter can be tolerated, as shown in a few examples in Figure S4. It is worth noting that the spectrum reported in Figure 3B acquired on the TAZ4 construct looks superficially similar to the overlay of the two different spectra reported in Figure 1B acquired on TAZ4 and ID4; importantly the former spectrum encodes the information on which domain the signals originate from, while still being part of the same protein construct. A further step was made by introducing the inversion recovery block before more complex experiments such as 3D HNCO and 3D HNCA experiments (3D IR-HNCO and 3D IR-HNCA respectively), using the BEST variant.^22^ The 3D IR-HNCO spreads the cross peaks in an additional indirect dimension (^13^C’) and can be used to resolve possible overlaps in the 2D IR-HN spectra still encoding the sign of cross peaks linked to local motional properties. The 3D IR-HNCA, a useful experiment for the sequence specific resonance assignment, is shown as an example in Figure 3D,E by reporting two ^1^H^N^-^13^C^α^ slices of the 3D spectrum extracted at 123.4 and 122.8 ppm in the ^15^N dimension. The pairs of signals associated with each ^1^H^N^ resonance (^1^H^N^_i_) allow to identify the resonances of two neighboring ^13^C^α^ nuclear spins (^13^C^α^_i_, ^13^C^α^_i-1_) in the sequence providing information for sequence specific assignment; their sign, encoded through the IR block inserted at the beginning of the pulse sequence, provides clean information about the local motional properties facilitating the sequence specific assignment process. In this version of the experiment, the sign of the signals constitutes additional information that suggests the domain of origin of the residue from which the peaks derive. The pulse sequences of all the inversion recovery experiments can be found as part of the Supporting Information. These experiments effectively complemented a set of NMR spectra, including both ^1^H-detected 3D HNCO,^23−25^ 3D HN(CA)CO,^25,26^ 3D CBCANH,^27^ 3D CBCA(CO)NH,^28,29^ 3D (H)N(CA)NNH,^30^ 3D (H)N(COCA)NNH,^31−33^ and ^13^C-detected 2D CACO,^34^ 2D CBCACO,^34^ 2D CCCO,^35^ and a 3D CBCACON,^36^ to obtain the sequence specific assignment of the TAZ4 resonances, including the Zn-ligands. Indeed, the Zn(II)-bound cysteine residues could be recognized based on the typical chemical shift of the ^13^C^β^ signals while the Zn(II)-bound histidine residues could be recognized by the typical pattern in a long-range 2D ^1^H-^15^NHSQC spectrum (Figure S5), that allows us to detect ^2^J_HN_ correlations as indicated in the figure. This spectrum shows that three histidine residues are bound to the Zn(II) ions through the N^ε^^2^ while four histidine residues are free. Other than the long-range 2D ^1^H-^15^N HSQC (Figure S5), we acquired a 2D ^1^H-^13^C TROSY^37^ and 2D (HB)CB(CGCD)HD^38^ to obtain the assignment of the histidine residues side chains.

### TAZ4 Construct Characterization

**Figure 4:**
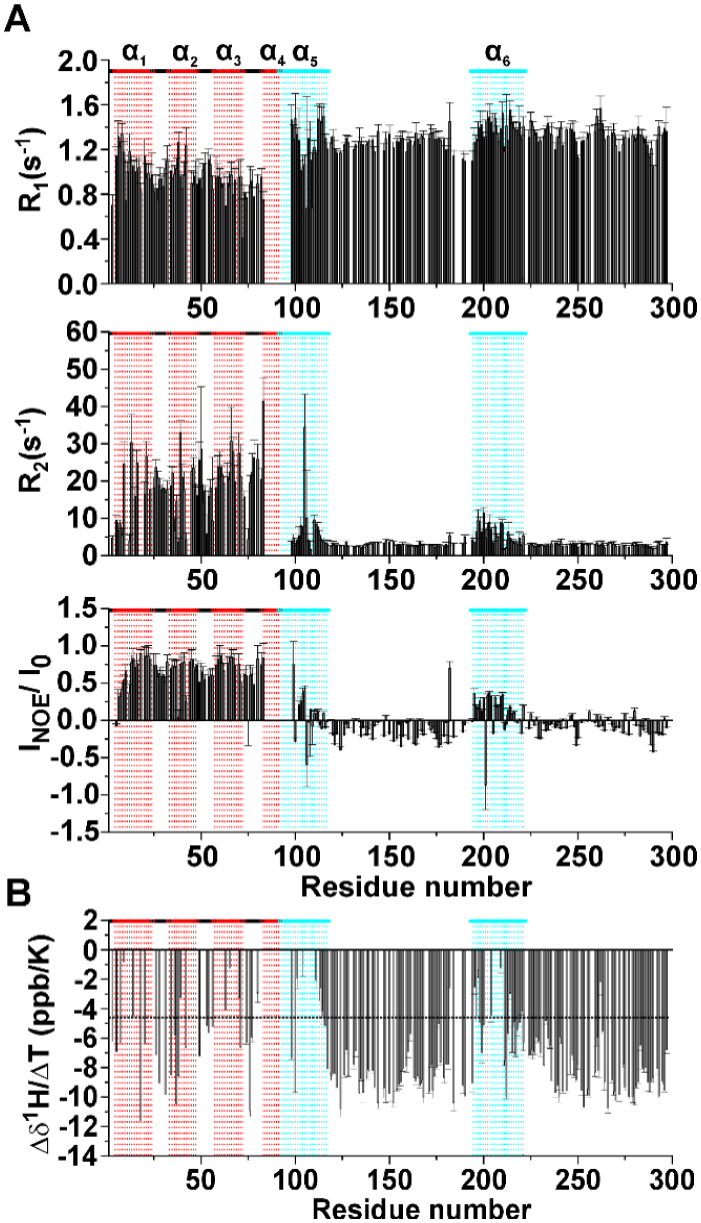
(A) ^15^N relaxation rates of TAZ4 at 298 K and 16.4 T (700 MHz). (B) Temperature coefficient (Δδ^1^H/ΔT) plotted against the residue number. Red boxes and drop lines indicate the α-helices of TAZ2, cyan boxes and drop lines indicate the partially folded helices described for ID4.

The dynamic properties of the TAZ4 construct were assessed determining the ^15^N relaxation rates and the heteronuclear ^1^H–^15^N NOEs (Figure 4A). ^15^N R_1_ values show an average higher value for the disordered region. ^15^N R_2_ and ^1^H–^15^N NOE values have very distinct patterns: for instance, the TAZ2 domain generally shows higher values than the ID4 domain. This is due to the higher flexibility of the IDR with respect to the globular region. The ^15^N relaxation values confirm the presence of the partially folded helices observed by Piai and coworkers for isolated ID4,^14^ also in this construct. These regions (highlighted in cyan in Figure 4A) are characterized by ^15^N R_2_ and ^1^H–^15^N NOE that are higher than the average values for the ID4 domain, two observations that indicate higher local rigidity.

Focusing on the TAZ2 domain, the NOE values are coherent with the structural and dynamic properties of a globular domain. The N-terminus of TAZ4 constitutes an exception, with smaller ^1^H–^15^N NOEs and ^15^N R_2_ values suggesting that being at the very beginning of the sequence these residues could be more flexible and less buried in the core of the globular domain as often observed for *N*-terminal and C-terminal residues within a protein construct. The rotational correlation time (τ_R_) obtained experimentally for TAZ2 in the TAZ4 construct is 11.6 ns, a value considerably larger of that expected for a small globular protein.^39,40^ For comparison we calculated the predicted τ_R_ value for TAZ2 by using HYDRONMR,^41^ obtaining a value of 7 ns. These data suggest that the presence of ID4 influences the mobility of the TAZ2 domain, either through exchange contributions to *R*_2_ or through reduced and more anisotropic tumbling by the adjacent disordered domain. The τ_R_ value of the TAZ2 domain in the TAZ4 construct is still smaller than the one predicted for a protein as large as TAZ4 (with calculated τ_R_ values well above 15 ns), meaning that this domain still possesses some rotational freedom.

**Figure 5:**
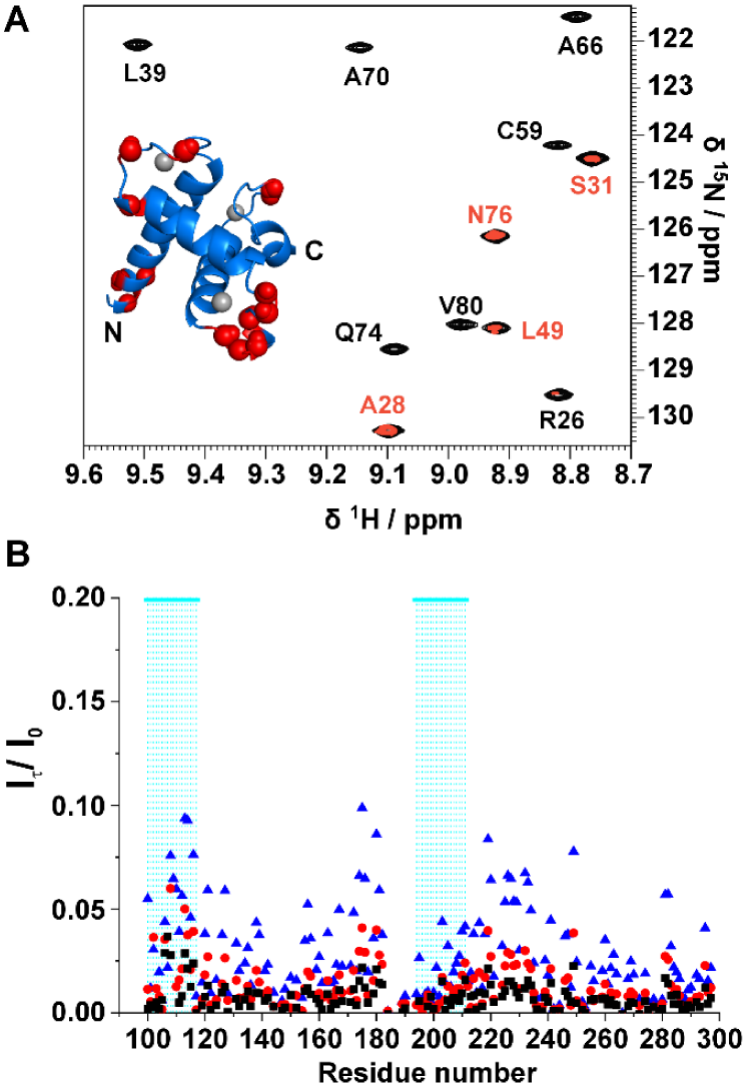
(A) superimposition of the same region of the ^1^H–^15^N HSQC reference spectrum of TAZ4 (black contours) with the CLEANEX spectrum acquired on the same sample with a delay of 100 ms (red contours) at 298 K and 21.1 T (900 MHz). A model of the TAZ2 domain is shown (PDB 1F81). Gray spheres represent Zn(II) ions; red spheres represent residues which give rise to a cross peak in the CLEANEX experiment demonstrating the presence of chemical exchange with the solvent protons. (B) Ratios of peak intensities between CLEANEX spectra and the reference spectrum plotted against the residue number for the ID4 region in TAZ4 recorded at 298 K and 21.1 T (900 MHz). The ratios are reported for the following delays: 10 ms (black squares), 20 ms (red circles) and 50 ms (blue triangles).

Changes of chemical shift of amide protons with temperature can give insights on the presence of intramolecular hydrogen bonds.^42^^1^H^N^ chemical shift differences at various temperatures were divided by the difference in temperature to obtain the temperature coefficient reported in Figure 4B as a function of the residue number. The horizontal black dashed line at −4.6 ppb/K represents the threshold above which the presence of H-bond is predicted with a probability of 85%.^42^ In the TAZ2 domain there is a modulation of the values of temperature coefficients that is expected from a fold constituted by α-helices connected through several external loop regions. This modulation derives from the different length of the hydrogen bonds that the amide protons form with surrounding residues.^42^ On the other hand, the disordered regions show uniformly negative values of temperature coefficients (below −4.6 ppb/K) that indicate the absence of intramolecular hydrogen bonds. The regions 98–117 and 194–221 show values that are above the threshold. These results, in line with what was observed through ^15^N relaxation rates, confirm the presence of the two partially formed helices α_5_ and α_6_. To establish the extent of solvent exchange at the residue level we performed the CLEANEX^43^ experiment with four values of mixing time: 10, 20, 50, and 100 ms. As mentioned above when discussing the ^15^N relaxation rates, there is a clear difference between the two domains of the protein. Amide protons of the globular domain poorly exchange with the solvent protons, except those of residues in the N-terminal region and in the exposed loops (Figure 5A). Amide protons of intrinsically disordered regions, on the other hand, are well exposed to the solvent, as described by the large exchange rates measured with the CLEANEX experiment, with lower effects observed for the two partially populated α-helices (Figure 5B).

### Are the Two Domains Independent?

**Figure 6:**
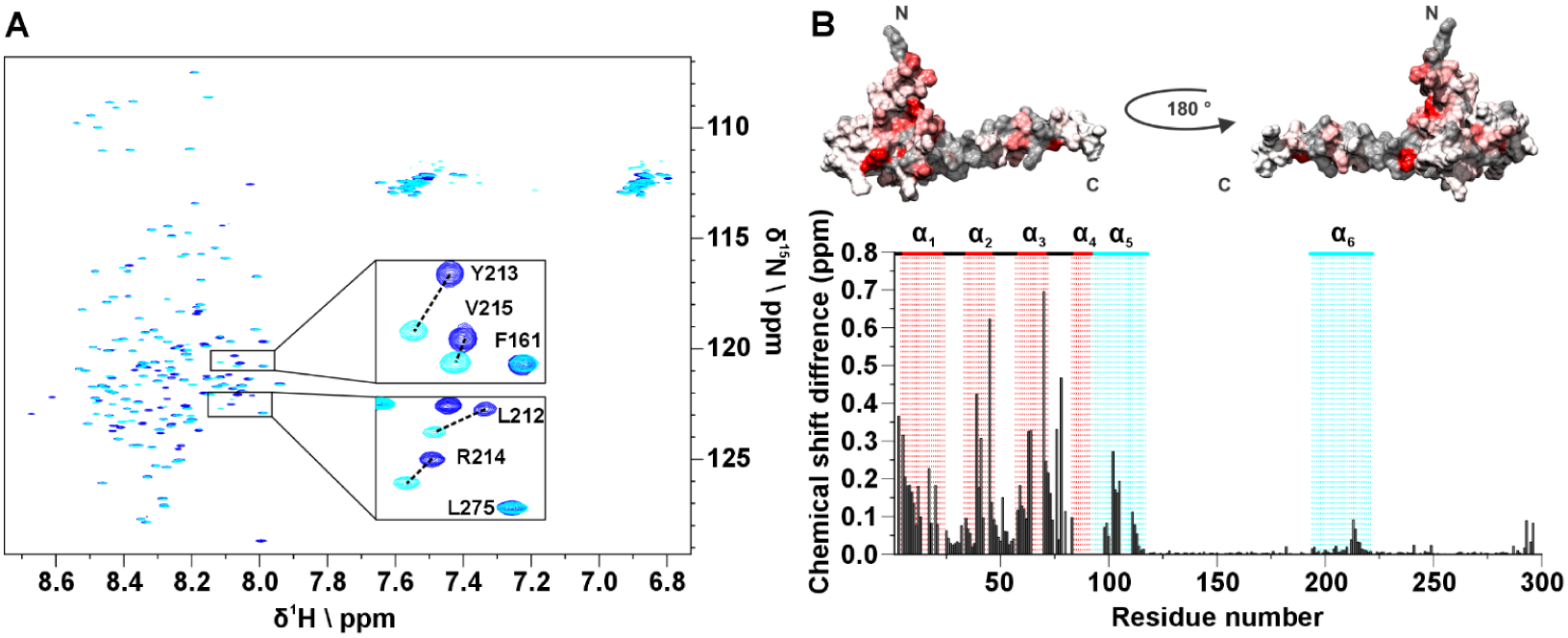
(A) Superimposition of 2D HN spectra of ID4 (blue contours) and TAZ4 (cyan contours). In the insets two regions are enlarged to show the shift experienced by some α_6_ residues’ amide protons. The spectra were recorded at 298 K and 16.4 T (700 MHz). The spectra are shown with the same contour levels, thus in the TAZ4 spectrum only the peaks belonging to the disordered region are visible. (B) Absolute values of the combined ^1^H and ^15^N chemical shift differences of TAZ4 with respect to chemical shifts observed for the two isolated domains (ID4 and TAZ2) are shown. On top of the figure the differences are mapped with colors on the TAZ2 structure (PDB 1F81) with increasing values from white to red. Information for the gray residues is not available. On the bottom of the figure the chemical shift difference is plotted for the entire construct against the residue number.

The presence of two domains within the same construct could lead to changes in their structural and dynamic properties with respect to the isolated ones. To test whether this is the case for TAZ4 we acquired a 2D HN experiment of ID4 in the same conditions as TAZ4 to compare the chemical shifts of the isolated domain to that of the ID4 domain within the TAZ4 construct. Figure 6A shows the superimposition of the two spectra. Generally, the disordered residues show minor perturbations, while measurable changes are observed for the residues belonging to the partially populated helices (Figure S6A); intensity changes are reported in Figure S6B. Specifically, the peaks of the residues belonging to α_5_ broaden and shift, while the peaks of the residues of α_6_ shift with a less pronounced broadening. Many residues of the TAZ2 domain within the TAZ4 construct are also perturbed by the presence of ID4 (the four mutated cysteine residues as well as the contiguous residues were not considered in the comparison made with respect to the available chemical shift data BMRB entry 4789).^7^ In Figure 6B the structure color code is based on the magnitude of the chemical shift changes, increasing from white to red. Notably, the residues that change the most are those of α_2_ and α_3_ residues facing α_4_, and those at the beginning of α_5_. These changes, partly expected considering that this is the region connecting the two isolated domains, are quite large and extend more than just a few residues, indicating a substantial change in this region. On the bottom of the panel, the chemical shift differences are plotted against the residue number for the entire TAZ4 construct.

We calculated the Secondary Structure Propensity (SSP) for both TAZ4 and ID4 to investigate possible changes in the propensity to sample the helical conformation (Figure S7). In addition, we compared the ^15^N relaxation properties of TAZ4 (Figure S8) with those already published for isolated ID4.^14^ The results show that while α_5_ seems more stabilized by the presence of the folded domain; for α_6_, both the SSP and the ^15^N relaxation properties do not change much and are not correlated with a different propensity to form an α-helix. These observations suggest that the chemical shift variations are due to interactions between the two domains, involving amino acids belonging to the second helix of ID4. For comparison CLEANEX experiments were repeated on the isolated ID4 domain in the same experimental conditions (Figure S6C,D): interestingly larger solvent exchange effects are monitored in absence of the TAZ2 domain.

### How Far Do Different Structural Models Reproduce NMR Observations?

**Figure 7:**
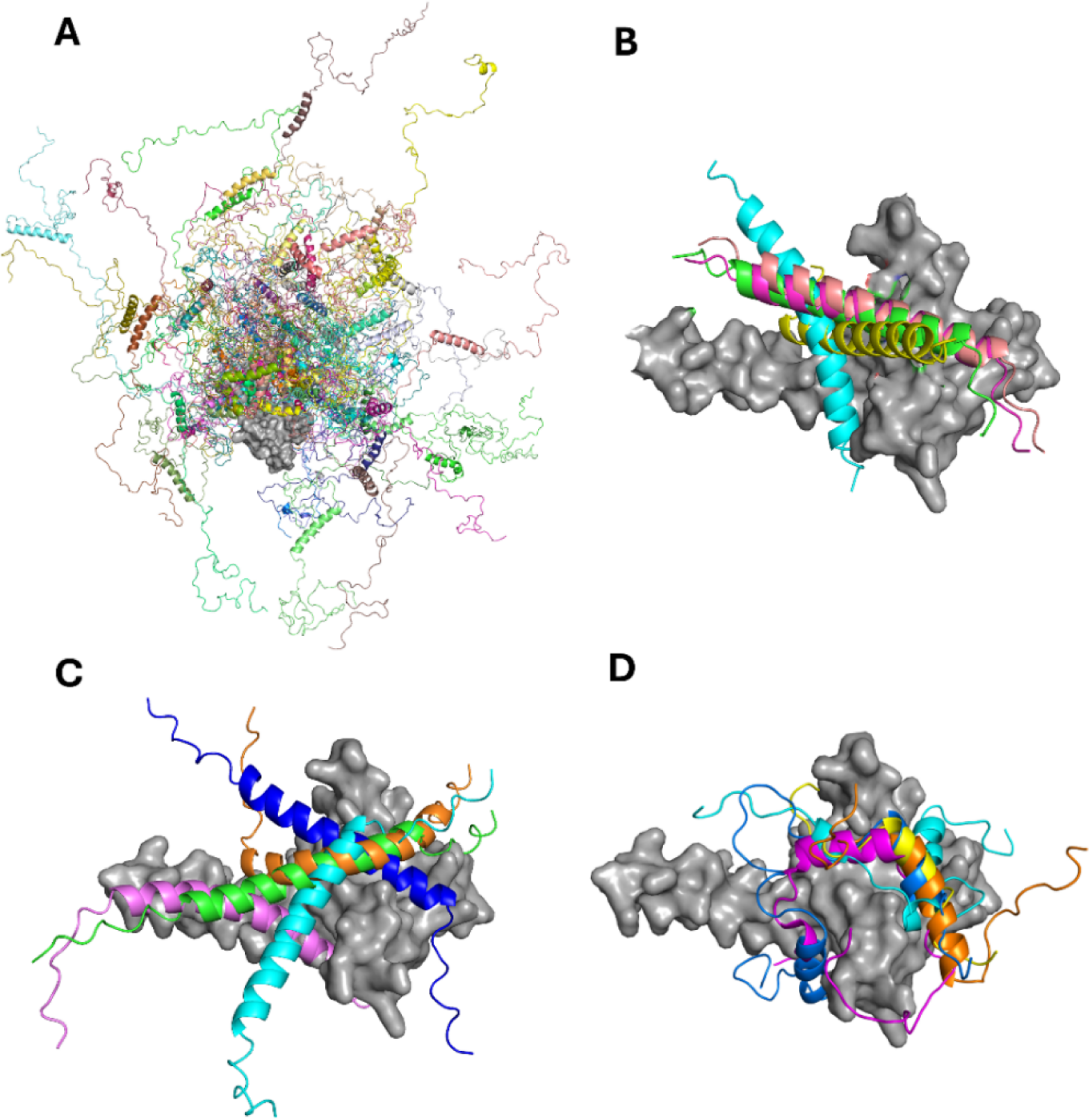
(A) 50 conformers generated by IDPConfGen aligned on the TAZ2 domain (gray surface). (B) Five structures of the whole TAZ4 construct generated by AlphaFold 3 aligned on the TAZ2 domain (gray surface). Colored ribbons represent α_6_ helices of each structure. The rest of the ID4 region was omitted from the figure for clarity. (C) AlphaFold 3 generated structures of the interaction between ID4 and TAZ2. Five structures are aligned on TAZ2 (gray surface). Colored ribbons represent α_6_ helices of each structure. The rest of ID4 was omitted from the figure for clarity. (D) Activation domain of IDPs (colored ribbons) interacting with TAZ2 (gray surface). The shown protein fragments are the activation domain of E1A (cyan), the activation domain 1 of E2A (orange), the transactivation domain of P53 (blue) and the transactivation domain of STAT1 (magenta).

Describing a complex protein construct as the one investigated in the present case is not an easy task, but it is important to visualize the results of experimental investigations through meaningful structural models. To this end, we compared our experimental results with the ensemble of conformers obtained through two different computational approaches: IDPConformerGenerator (IDPConfGen)^44^ and AlphaFold 3 (AF3)^45^ (Figure 7). IDPConfGen allows us to visualize the conformational space sampled by TAZ4. For the calculation, the Local Disordered Region Sampling (LDRS) module^46^ was used. This module allows the modeling of the backbone of disordered regions that are next to a globular domain. The algorithm generated the ID4 conformers by taking the dihedral angles from a custom subset of PDB structures, sampling both loops and α-helices. Then, it connected these conformers to the C-terminus of TAZ2 without modifying its structure. IDPConfGen nicely shows the disorder (and high flexibility) of the ID4 domain in the TAZ4 construct, an aspect in agreement with our NMR experimental data (Figure 7A).

AF3 models the 3D structure starting from the sequence, building the whole construct. In these models, the TAZ2 domain is very similar to the X-ray structure of p300-TAZ2^47^ (RMSD = 1.64 Å). α_5_ is modeled as fully populated and fused with α_4_. α_6_ is predicted to be fully populated too. In the crystal structure obtained by Miller and coworkers^47^ of the TAZ2 domain of p300, which shows an identity of 89.5% and a similarity of 94.7% with the corresponding domain of CBP (Figure S9), the fourth helix of the construct is extended to contain the first 23 residues of the ID4 region. Dyson and Wright^48^ proposed that this elongated helix is stabilized in the crystals by lattice contacts. The data reported in the present work suggests that in solution and in the presence of the entire ID4 region, this long helix is populated, even if the occurrence of conformational exchange makes the relevant NMR signals difficult to detect. Interestingly, AF3 predicts the existence of an interaction between α_6_ and the TAZ2 domain (Figure 7B), as suggested by the NMR data. We repeated the calculations considering TAZ2 and ID4 as separated domains and the software predicts the interactions also in this case, with different poses for the ID4 helix (Figure 7C). The lack of a well-defined interaction pocket suggested by AF3 is consistent with the chemical shift perturbations shown in Figure 6B. The picture that appears from this analysis is of a highly dynamic construct that samples a large conformational space, with the presence of a transient crosstalk between the folded domain and the disordered region.

## Discussion

### On IDR/TAZ2 Interactions

In the literature, several studies have explored the interactions between the TAZ2 domain and the activation domains of various disordered proteins (Figure 7D). TAZ2 has been shown to interact with the transactivation domains of p53,^49^ which is involved in tumor suppression and cellular stress responses. Additionally, TAZ2 interacts with the first activation domain of E2A,^50^ which is involved in the transcriptional activation during lymphocyte development, and with E1A oncoprotein,^51^ which is involved in the activation of the viral gene expression and in the deregulation of cellular processes. The interaction with signal transducer and activator of transcription 1 (STAT1) has also been reported.^52^ The interactions with all these partners typically involve the hydrophobic region localized at the interface of the first three α-helices of TAZ2. Even though these disordered proteins do not share a sequence homology, all of them fold upon binding when interacting with TAZ2, forming amphipathic helices. The cleft present on TAZ2 shows chemical shift changes when part of the TAZ4 construct, suggesting the presence of transient interactions with α_6_ that is constituted by both hydrophobic and negatively charged residues. The latter could thus play a role in protecting this interaction site until stronger binders, such as the examples mentioned above, are present. IDRs acting as regulatory effectors with autoinhibitory and/or allosteric mechanisms have been reported in literature.^53,54^ For instance, it has been shown that IDRs are involved in fine-tuning of enzymatic activity^55^ and DNA binding affinity^56^ through intramolecular interactions. In this framework, ID4 could modulate the interactions of TAZ2 with its protein partners. An example of regulation by competitive binding between two disordered protein regions has been described for the TAZ1 domain of CBP.^57^ A similar mechanism could exist for TAZ2, for which only proteins with a higher affinity than ID4 can successfully interact with the folded domain. Interactions involving disordered proteins occurs with different mechanisms^58^ including folding upon binding and the formation of fuzzy complexes.^59^ Our data suggest that the secondary structure properties of ID4 does not change substantially upon interaction with TAZ2, in contrast to what happens for the TAZ2 partners mentioned above. Further investigation is needed to characterize the regulatory mechanism that we suggest exists for ID4 in the context of the TAZ4 construct of CBP.

### A Novel NMR Approach to Study Highly Flexible Multidomain Proteins

The complexity of NMR spectra of highly flexible multidomain proteins can be overcome by “filtering/editing” different subsets of signals on the basis of their NMR properties. In general, it is not difficult to select NMR signals originating from highly flexible regions, characterized by lower transverse relaxation rates with respect to NMR signals originating from globular domains. The smaller relaxation rates allow one to select disordered regions just by including a transverse relaxation filter that suppresses faster relaxing signals, such as those belonging to the globular domain itself. An elegant experiment that can be included in this category is the CON experiment (Figure 1C), in which the two coherence transfer elements naturally included in the pulse sequence can act as a filter. Thus, the CON experiment provides highly resolved NMR spectra that reveal in a clean way signals originating from the highly flexible regions also when they are part of more complex protein constructs (Figure 1C).^20^ The method proposed here, instead, exploits ^1^H longitudinal relaxation enhancement (LRE) through band-selective inversion recovery of amide protons^21,60−62^ to facilitate detection of the signals deriving from nuclei of residues in more structured portions of a protein. In our construct the most structured residues and the highly flexible ones are segregated in two different domains, even if some residues belonging to the TAZ2 domain are flexible (e.g., the loops) and some residues of the disordered region are quite rigid (e.g., the partially folded helices). This allows the almost complete selection of signals of the globular domain at the expense of those of the disordered region. The contribution to the ^1^H LRE deriving from ^1^H–^1^H cross-relaxation increases with increase of local effective correlation time and thus favors globular domains with respect to highly flexible disordered ones, in the experimental conditions used in the present work. Indeed, the exchange of amide protons with water is hampered at pH 5.5, thus minimizing the most efficient contribution to longitudinal recovery of amide protons in IDRs.^61^ Moreover, the active use of LRE permits a reduction of the experimental time. Specifically, when filtering/editing on the basis of longitudinal relaxation, long recovery delays are necessary to ensure complete recovery of the magnetization to the equilibrium state. These delays become shorter when exploiting longitudinal relaxation enhancement techniques. The approach, once tested through 1D experiments to assess its feasibility, was used to determine the inversion recovery properties of the signals deriving from the different protein domains (Figures 2B and S3). This provided a residue-specific measurement of the recovery profile of amide protons confirming the validity of the approach. Selection of a specific inversion recovery delay for which the polarization of the signals of the disordered domain still has negative values while those of nuclear spins in the globular domain are positive, allowed us to readily identify in simple 2D NMR spectra the signals with different relaxation properties and thus associate them to a specific protein domain. In some cases, it may be beneficial to record multiple data sets tailored to different sets of signals. Tests performed at different magnetic fields clearly show that the method is generally applicable with minimal adjustments (Figure S10). Should the interest shift mainly to the study of globular domains in multidomain protein constructs the delay can also be optimized to reduce as much as possible the intensities of signals of IDRs, a solution that also has the advantage of reducing T_1_ noise in the spectra. The method is easily adaptable to any multidimensional pulse sequence, such as the 3D HNCO and HNCA described here. The resulting 3D spectra have been shown to be critical in obtaining the sequence-specific resonance assignment of TAZ4, resulting in the sequence specific assignment of 90% of the expected signals, with only the first residues of ID4 missing.

It is worth comparing the approach presented here with other possible strategies useful for distinguishing ordered and disordered protein regions. The heteronuclear ^1^H–^15^N NOE experiment provides the first observables that allow to discriminate between regions of proteins characterized by very different local dynamics, as discussed above for TAZ4 and shown in Figures 4 and S11. However, its low sensitivity makes it challenging to be implemented into more complex NMR experiments, such as 3D triple resonance experiments necessary for assignment purposes. The other observables that provide complementary information to discriminate between globular and disordered regions are transverse relaxation rates (^15^N *R*_2_, Figure 4) as well as solvent exchange processes measured with experiments like the CLEANEX used for TAZ4 (Figure 5). These observables can be easily used to select signals originating from disordered regions at the expense of those of the globular domain. The orthogonal selection, i.e., the selection of the signals of the globular domain, could in principle be obtained through difference spectra, that is subtracting signals of the disordered regions from spectra containing all signals. However, the sharpness of the signals of residues in disordered regions often prevents a clean selection of the broader signals of the globular one. A different strategy to discriminate between ordered and disordered protein regions, consists in varying two factors, temperature and pH, that strongly influence exchange processes with solvent protons. Solvent exposed residues undergo relevant changes under varying the external conditions (T, pH), often leading to signal broadening beyond detection, whereas residues within globular cores remain largely unaffected. On these grounds, increasing temperature and pH can be used to suppress signals from solvent exposed protein regions while maintaining signals from globular cores.^63^ The 2D HN NMR spectra of TAZ4, acquired under different temperature and pH conditions as shown in Figure S12, clearly demonstrate this trend. As expected, the signals from the disordered regions are reduced in intensity, although not completely. However, external loops of the globular domain also exhibit signal broadening beyond detection with increasing pH as shown in the insets of Figure S12. Moreover, the dependence of chemical shifts on these two parameters (T, pH) complicates the unambiguous identification of resonances under different conditions, often necessitating additional sequence-specific resonance assignment experiments to confirm assignments in different conditions (T, pH). This is particularly challenging when focusing on complex systems as multidomain proteins. The method proposed here provides a tool that not only avoids the need for reassignment of resonances but also aids in this process by distinguishing cross-peaks based on their sign, which depends on the dynamic properties of the residue which it originates from.

A comment is due on the effect of temperature and pH on the longitudinal relaxation enhancement filter proposed. It is well-established that in conditions in which exchange processes with the solvent are modest, the major contributions to longitudinal relaxation enhancement derives from ^1^H–^1^H dipolar cross relaxation rates, which in turn depend on the local correlation time, the underlying rational of the proposed approach. However, as solvent exchange processes become more effective, these also contribute to longitudinal relaxation enhancement and in extreme conditions they may become so efficient to reverse the situation: solvent exposed amides may become the signals that experience faster recovery with respect to signals of nonexchangeable protons. This could be in principle an orthogonal approach to achieve discrimination between two sets of signals. However, often many ^1^H^N^ signals are broadened beyond detection in these conditions reducing the information content. Therefore, the initial screening to maximize ^1^H^N^ signal detectability for the investigation of an heterogeneous protein also leads to optimal conditions for the implementation of the longitudinal relaxation enhancement filter proposed here. Since the two most relevant parameters that modulate solvent exchange rates (temperature and pH) can be independently changed, it is generally possible to find optimal conditions (Figure S13).

Longitudinal relaxation filters have been used since the early days of NMR, for example to investigate paramagnetic systems in which the nuclear spins in the proximity of the paramagnetic center experience additional contributions to relaxation with respect to the diamagnetic ones (both selective and nonselective inversion recovery experiments were used).^64,65^ In the present case we opted for a novel approach based on exploiting longitudinal relaxation enhancement effects to capitalize on the different relaxation behavior of ordered and highly flexible protein regions. The differences in local dynamics have been also used in MAS-solid-state NMR experiments to distinguish more rigid, ordered protein regions from highly flexible disordered ones by exploiting either cross-polarization or scalar couplings in NMR experiments.^66,67^

Recently, a T_1_-filtered DEER EPR pulse sequence has been proposed to deconvolute the distance distribution obtained from a mixture of monomeric and oligomeric proteins.^68^ This experiment presents an electron inversion recovery pulse followed by a variable recovery delay prior to the DEER sequence. The peak intensities in the distance distribution depend on the duration of the variable delay, thus it is possible to assign them to the different species present in the sample relying on their apparent T_1_.

## Conclusions

In summary, this work used NMR spectroscopy to explore the multidomain protein TAZ4, with the primary goal of developing an experimental strategy for studying multidomain proteins. The designed approach provided critical information regarding the protein’s structure and dynamics, as well as key information for sequential assignment of this heterogeneous construct. The chemical shift perturbations observed when comparing the entire construct to the individual domains in isolation demonstrate the existence of an interaction between the two sections of the construct, and several other NMR observables aid in describing the intricate nature of these interplays. The methodology developed in this study holds potential for broader application in the investigation of other biologically relevant multidomain proteins, but also advanced our knowledge of TAZ4 properties, providing the groundwork for further exploration into the realm of CBP.

## Supporting Information Available

Experimental methods, pulse sequences of the inversion recovery experiments, ^1^H^N^ longitudinal relaxation rates, comparison of recovery delays in 2D IR HN experiments, long-range 2D ^1^H–^15^N HSQC spectrum on histidine side chains, secondary structure propensity calculation, ^15^N relaxation rates at 283 K, intensity ratios of CLEANEX experiments recorded on ID4, comparison of signal intensities of ID4 and TAZ4, sequence alignment between P300-TAZ2 and CBP-TAZ2, comparison of 2D IR HN spectra at different magnetic fields, effects of pH and temperature on TAZ4 2D HN spectrum, and 2D IR HN spectrum recorded at pH 6.5 and 283 K (PDF)

## Accession Codes

TAZ4 assigned chemical shifts have been deposited into the BMRB repository (BMRB ID 52655).

## Abbreviations

NMR: nuclear magnetic resonance
CBP: CREB binding protein
IR: inversion recovery
IDP: intrinsically disordered protein
IDR: intrinsically disordered region
1D: one dimension
2D: two dimensions
3D: three dimensions
