# Supplementary material for: Decoding Order and Disorder in Proteins by NMR Spectroscopy
Source: J Am Chem Soc. 2025 Apr 14;147(16):13146–57. doi: 10.1021/jacs.4c14959 (PMC12022988; doi:10.1021/jacs.4c14959)
Supplement: Supplementary file 1 — ja4c14959_si_001.pdf [file ja4c14959_si_001.pdf]

Supporting Information

for

Decoding order and disorder in proteins by NMR spectroscopy

Lorenzo Bracaglia, Silvia Oliveti, Isabella C. Felli*, Roberta Pierattelli*

Department of Chemistry "Ugo Schiff" and Magnetic Resonance Center, University of Florence, Sesto Fiorentino, Italy

CONTENT:

1. **Methods**
    a. **Sample Preparation**
    b. **NMR spectroscopy**
    c. ***In silico* calculations**
2. **Supplementary figures**
3. **Pulse sequences**
4. **Bibliography**

1. Methods
a. Sample preparation

The protein is expressed with some difficulty in *E. coli* cells and with a relatively low yield. This is due to the construct degradation after induction with IPTG. To overcome this issue, we adopted a strategy that aimed to maximize the number of cells [1] and to reduce the induction time, thus preventing degradation. We refer to this protocol as "High-OD". As detailed below, cell cultures are induced with IPTG at OD_{600} around 5 and the induction time is only 2 hours long (at 30 °C). These growth conditions need to be supported with 10 g/L of glucose, which is higher than the concentration used in common expression protocols (e.g. 4 g/L). The yield of the protocol described in this work and that of Marley's protocol [2] were compared. The former gives a final yield of protein of 5 mg per liter of culture, while the latter only 1.5 mg. The high-OD protocol gives better results with respect to Marley's protocol even if we compare the ratio between the mass of purified protein and that of the ^{13}C-glucose used for cell growth.

The recombinant vector pET28a+ containing the human CBP-TAZ4-Δ4-cys (C1775A, C1783A, C1826A, C1827A) gene was transformed into *E. coli* BL21 (DE3) Gold strain for protein expression. For ^{15}N- and ^{13}C -^{15}N-labeled protein production, a colony from a freshly transformed plate was selected to inoculate in LB medium (0.6 L) containing 50 mg L^{-1} kanamycin and was grown overnight at 27°C and 180 rpm. Cells from the overnight LB cultures were collected via centrifugation (15 minutes at 2500 rpm). The pellet was resuspended into the same volume of M9 minimal medium (25 mM KH_2PO_4, 50 mM Na_2HPO_4, 10 mM NaCl, 0.2 mM $CaCl_2$, 5 mM $MgSO_4$, 1 µg L^{-L} each of biotin and thiamine, 1 g/L $(^{15}NH_4)_2SO_4$, and 10 g/L mM glucose/$^{13}C_6$-glucose) and divided in 4 x 150 mL cultures that were grown at 30°C with constant agitation at 160 rpm for 1.5 hours. $ZnCl_2$ was added to a final concentration of 0.1 mM prior to the induction. The cells were induced with 0.5 mM isopropyl β-D-1-thiogalactopyranoside and allowed to grow for an additional 2 hours at 30°C. The culture was then harvested at 8000 rpm for 20 min and the pellet was stored at −20°C. Frozen cells were thawed and suspended in 80 mL of equilibration buffer A (20 mM TRIS, 40 mM imidazole, 500 mM NaCl, 1 mM DTT, 0.1 mM $ZnSO_4$, pH 8). Cells were disrupted by sonication on ice with cycles of 10 s with 2 s delay pulses for 25 min. Lysed cells were centrifuged at 40,000 rpm for 40 min at 4°C. Affinity chromatography was performed on a 5 mL HisTrap FF column (GE Healthcare) preequilibrated with buffer A. A linear gradient between buffer A and buffer B (buffer A with 0.5 M imidazole) was applied for 20 column volumes. TAZ4 was eluted with an imidazole concentration of 100 mM. Fractions were analyzed by SDS-PAGE, concentrated to reach the desired concentration and stored at -80 °C.

b. NMR Spectroscopy

All the experiments were recorded on a sample of 250 µM ^{13}C-^{15}N uniformly labelled TAZ4 in 25 mM sodium acetate buffer, 50 mM sodium chloride, 0.1 mM zinc chloride, 2 mM TCEP, 0.05% sodium azide, 10% deuterium oxide at pH 5.5, and on a sample of 300 µM ^{15}N uniformly labelled TAZ4 in the same buffer. The experiments were recorded on four different spectrometers at 298 K (if not specified):

- Bruker Ascend NEO spectrometer operating at 1200.64 MHz ^{1}H, 301.91 MHz ^{13}C and 121.67 MHz ^{15}N frequencies, equipped with a cryogenically cooled probe head optimized for ^{13}C direct detection (CPTXO). Hereafter this will be referred to as 1200 MHz spectrometer.
- Bruker Avance NEO spectrometer operating at 899.78 MHz ^{1}H, 226.27 MHz ^{13}C and 91.19 MHz ^{15}N frequencies, equipped with a cryogenically cooled probe head (CPTCI). Hereafter this will be referred to as 900 MHz spectrometer.
- Bruker Avance NEO spectrometer operating at 700.06 MHz ^{1}H, 176.05 MHz ^{13}C and 70.94 MHz ^{15}N frequencies, equipped with a cryogenically cooled probe head optimized for ^{13}C-direct detection (CPTXO). Hereafter this will be referred to as 700 MHz spectrometer.
- Bruker Avance NEO spectrometer operating at 700.13 MHz ^{1}H, 176.07 MHz ^{13}C and 70.95 MHz ^{15}N frequencies, equipped with a cryogenically cooled probe head (CPTCI). Hereafter this will be referred to as 700 MHz spectrometer 2.

Assignment

3D (H)N(CA)NNH[3] and 3D (H)N(COCA)NNH[4–6] experiments were performed on the 1200 MHz spectrometer. The carrier frequencies for ^{1}H and ^{15}N were 4.7 ppm and 117 ppm, respectively. Surbop90 and Surbop180 shapes[7] of durations of 333 µs, were employed for ^{13}C band-selective π/2 and π flip angle pulses. Decoupling of ^{15}N was achieved with Garp4 (250 µs) decoupling sequence.

2D CBCACO[8,9], 2D CACO[9,10], 2D CON[10] and 2D CCCO[9,10] ^{13}C detected experiments were recorded on the 700 MHz spectrometer. CBCACO spectra were acquired with a relaxation delay of 1.2 s, 32 scans and a spectral width of 28.4

ppm x 80 ppm (1200 x 600 points). The carrier frequencies were set to 173.5 ppm for C′, 53.2 ppm for C^{α}, and 43 ppm for $C^{\alpha/\beta}$. For ^{13}C band-selective $\pi/2$ and π flip angle pulses Q5_sebop (or time reversed Q5_sebop) and Q3_surbop shapes[11] of duration of 300 μs and 230 μs respectively were used. Decoupling of ^{1}H and ^{15}N was achieved with waltz65 (100 μs) and garp4 (250 μs) decoupling sequences, respectively. All gradients used had a smoothed square shape. For each increment of the experiment the in-phase (IP) and antiphase (AP) components were recorded and properly combined to achieve IPAP[12] virtual decoupling.

2D CACO[6,7] spectrum was acquired with a relaxation delay of 1.2 s, 16 scans and a spectral width of 28.4 ppm x 49.8 ppm (1200 x 512 points). The carrier frequencies were set to 173.5 ppm for C′, 53.2 ppm and 46 ppm for $C^{\alpha/\beta}$. For ^{13}C band-selective $\pi/2$ and π flip angle pulses Q5_sebop (or time reversed Q5_sebop) and Q3_surbop shapes[11] of duration of 300 μs and 230 μs respectively were used. Decoupling of ^{1}H and ^{15}N was achieved with waltz65 (100 μs) and garp4 (250 μs) decoupling sequences, respectively. All gradients used had a smoothed square shape. For each increment of the experiment the in-phase (IP) and antiphase (AP) components were recorded and properly combined to achieve IPAP[12] virtual decoupling.

2D CON[8] spectrum was acquired with a relaxation delay of 2 s, 8 scans, and a spectral width of 28.4 ppm x 45 ppm (1024 x 512 points). The carrier frequencies were set to 173.5 ppm for C′, 53.2 ppm for C^{α}, and 123 ppm for ^{15}N. For ^{13}C band-selective $\pi/2$ and π flip angle pulses Q5_sebop (or time reversed Q5_sebop) and Q3_surbop shapes of duration of 300 μs and 230 μs respectively were used. A low power $\pi/2$ flip angle pulse of 25 μs was used for the FLOPSY16 sequence[13]. Decoupling of ^{1}H and ^{15}N was achieved with waltz65 (100 μs) and garp4 (250 μs) decoupling sequences, respectively. All gradients used had a smoothed square shape. For each increment of the experiment the in-phase (IP) and antiphase (AP) components were recorded and properly combined to achieve IPAP virtual decoupling.

^{13}C detected 2D CCCO[6,7] spectrum was acquired with a relaxation delay of 1.2 s, 32 scans, and a spectral width of 28.4 ppm x 70.1 ppm (1200 x 700 points). The carrier frequencies were set to 173.5 ppm for ^{13} C′, 53.2 ppm for $C^{\alpha,}$ 43 ppm for $C^{\alpha/\beta}$. For ^{13}C band-selective $\pi/2$ and π flip angle pulses Q5_sebop (or time reversed Q5_sebop) and Q3_surbop shapes of duration of 300 μs and 230 μs respectively were used. Decoupling of ^{1}H and ^{15}N was achieved with waltz65 (100 μs) and garp4 (250 μs) decoupling sequences, respectively. All gradients used had a smoothed square shape. For each increment of the experiment the in-phase (IP) and antiphase (AP) components were recorded and properly combined to achieve IPAP virtual decoupling.

3D IR HNCA experiment was acquired on the 900 MHz spectrometer. This experiment is based on the BEST variant of the HNCA experiment[14]. The spectrum was acquired with a relaxation delay of = 2 s, a spectral width of 13.9 ppm x 28 ppm x 34 ppm (2048 x 66 x 128 points) and 16 scans. The carrier frequencies were set to 4.7 ppm for ^{1}H, 53.2 ppm for C^{α} and 117 ppm for ^{15}N. The selective inversion of the amide protons region was achieved with a 1200 μs Reburp shaped pulse centered at 8.3 ppm. A rectangular z gradient (1%) was applied during the inversion recovery delay of 150 ms. PC9 and Eburp2 (or time reversed Eburp2) shapes of duration of 1425 μs and 1075 μs respectively, were employed for ^{1}H band-selective $\pi/2$ flip angle pulses. Reburp shape of duration 908 μs was used for ^{1}H band-selective π flip angle pulse. Bip-720-50-20 shape of duration 133 μs was used for ^{1}H broadband inversion. For ^{13}C band-selective $\pi/2$ and π flip angle pulses G4 (or time reversed G4) and Q3 shapes of duration of 273 μs and 171 μs respectively were used. ^{15}N decoupling was achieved with garp4 (350 μs) decoupling scheme. The ^{13}C band-selective pulses on C^{α} and C′ were applied at the center of each region respectively. All gradients used had a smoothed square shape.

3D IR HNCO was acquired on the 700 MHz spectrometer. This experiment is based on the BEST variant of the HNCO experiment[14]. The spectrum was acquired with a relaxation delay of 1.45 s, 16 scans and a spectral width of 13.7 ppm x 35 ppm x 14 ppm (1024 x 64 x 128 points). The carrier frequencies were set to 4.7 ppm for ^{1}H, 173.5 ppm for C′ and 117 ppm for ^{15}N. The selective inversion of the amide proton region was achieved as in the previous experiment. PC9 and Eburp2 (or time- reversed Eburp2) shapes of 1830 μs and 1415 μs respectively, were employed for ^{1}H band-selective $\pi/2$ flip angle pulses. Reburp shape of 1165 μs was used for ^{1}H band-selective π flip angle pulse. Bip-720-50-20 shape of duration 170 μs was used for ^{1}H broadband inversion. ^{15}N decoupling was achieved with garp4 (350 μs) decoupling scheme. The ^{13}C band-selective pulses on C^{α} and C′ were applied at the center of each region respectively. All gradients used had a smoothed square shape.

3D HNCO[15–17] was acquired on the 700 MHz spectrometer 2. The spectrum was acquired with a relaxation delay of 1 s, 8 scans and a spectral width of 13.7 x 28 x 12 ppm (2048 x 92 x 106 points). The carrier frequencies were set to 4.7 ppm for ^{1}H, 173 ppm for C′ and 117 ppm for ^{15}N. For ^{13}C band-selective $\pi/2$ and π flip angle pulses Q5_sebop (or time reversed Q5_sebop) and Q3_surbop shapes of duration of 300 μs and 231 μs respectively were used. ^{15}N decoupling was achieved with garp4 (250 μs) decoupling scheme. The ^{13}C band-selective pulses on C^{α} and C′ were applied at the center of each region respectively. All gradients used had a smoothed square shape.

3D HN(CA)CO[17,18] was acquired on the 700 MHz spectrometer 2. The spectrum was acquired with a relaxation delay of 1 s, 16 scans and a spectral width of 13.7 x 28 x 12 ppm (2048 x 92 x 106 points). The carrier frequencies were set to 4.7 ppm for ^{1}H, 173 ppm for C′ and 117 ppm for ^{15}N. For ^{13}C band-selective π/2 and π flip angle pulses Q5_sebop (or time reversed Q5_sebop) and Q3_surbop shapes of duration of 300 µs and 231 µs respectively were used. ^{15}N decoupling was achieved with garp4 (250 µs) decoupling scheme. The ^{13}C band-selective pulses on C^{α} and C′ were applied at the center of each region respectively. All gradients used had a smoothed square shape.

3D CBCANH[19] was acquired on the 700 MHz spectrometer 2. The spectrum was acquired with a relaxation delay of 1 s, 16 scans and a spectral width of 13.7 x 28 x 60.4 ppm (2048 x 92 x 128 points). The carrier frequencies were set to 4.7 ppm for ^{1}H, 41 ppm for $C^{\alpha\beta}$ and 117 ppm for ^{15}N. For ^{13}C band-selective π/2 and π flip angle pulses Q5_sebop (or time reversed Q5_sebop) and Q3_surbop shapes of duration of 300 µs and 231 µs respectively were used. ^{15}N decoupling was achieved with garp4 (250 µs) decoupling scheme. The ^{13}C band-selective pulses on C^{α} and C′ were applied at the center of each region respectively. All gradients used had a smoothed square shape.

3D CBCA(CO)NH[20,21] was acquired on the 700 MHz spectrometer 2. The spectrum was acquired with a relaxation delay of 1 s, 8 scans and a spectral width of 13.7 x 28 x 60.4 ppm (2048 x 92 x 128 points). The carrier frequencies were set to 4.7 ppm for ^{1}H, 41 ppm for $C^{\alpha\beta}$ and 117 ppm for ^{15}N. For ^{13}C band-selective π/2 and π flip angle pulses Q5_sebop (or time reversed Q5_sebop) and Q3_surbop shapes of duration of 300 µs and 231 µs respectively were used. ^{15}N decoupling was achieved with garp4 (250 µs) decoupling scheme. The ^{13}C band-selective pulses on C^{α} and C′ were applied at the center of each region respectively. All gradients used had a smoothed square shape.

3D ^{13}C detected CBCACON[22] was acquired on the 1200 MHz spectrometer. The spectrum was acquired with a relaxation delay of 1 s, 4 scans and a spectral width of 31.8 x 45.1 x 66.2 ppm (1800 x 132 x 228 points). The carrier frequencies were set to 173.5 ppm for ^{13} C′, 53.2 ppm for $C^{\alpha,}$ 43 ppm for $C^{\alpha/\beta}$ and 123 ppm for ^{15}N. For ^{13}C band-selective π/2 and π flip angles Surbop90 and Surbop180 pulses shapes of duration of 333 µs were respectively used. ^{1}H and ^{15}N decoupling was achieved with garp4 (105.75 µs) and p5m4sp180 (250 µs) decoupling schemes respectively. All gradients used had a smoothed square shape.

Histidine residues side chains

The $^{2}J_{HN}$ ^{1}H-^{15}N HSQC[23], the (HB)CB(CGCD)HD[24] and the ^{1}H-^{13}C TROSY[25] experiments for the characterization of the histidine side chains were acquired on the 900 MHz spectrometer. The HSQC experiment was acquired with a relaxation delay of 4 s, a spectral width of 29.9 ppm x 243.7 ppm (4096 x 1024 points) and 32 scans. The carrier frequencies for ^{1}H and ^{15}N were set to 4.7 ppm and 190 ppm respectively. The delay for the INEPT transfer was set to 10.6 ms, considering a heteronuclear $^{2}J_{HN}$ coupling constant of 23.5 Hz. Decoupling of ^{15}N was achieved with the Garp4 (250 µs) decoupling sequence.

2D ^{1}H-^{13}C-TROSY experiment was acquired with a relaxation delay of 1 s, a spectral width of 13.9 ppm x 40 ppm (2048 x 256 points) and 16 scans. The carrier frequencies for ^{1}H and ^{13}C were set to 4.7 ppm and 120 ppm respectively. The delay for the INEPT transfer was set to 1.8 ms, considering a heteronuclear $^{1}J_{HC}$ coupling constant of 140 Hz.

2D (HB)CB(CGCD)HD experiment was acquired with a relaxation delay of 1.5 s, a spectral width of 16.3 ppm x 40 ppm (2048 x 152 points) and 32 scans. The carrier frequencies for ^{1}H and ^{13}C were set to 4.7 ppm and 32 ppm respectively. G4 (or time reverse G4) and Q3 shapes of durations of 273 µs and 171 µs respectively, were employed for ^{13}C band-selective π/2 and π flip angle pulses, except for the π pulses that should be band-selective on the C^{β} region (Q3_surbop, 700 µs). Decoupling of ^{1}H and ^{13}C was achieved with Waltz65 (55 µs) and Garp (50 µs) decoupling sequences respectively. The ^{13}C band selective pulses on C^{aro} and C^{β} were given at the center of each region, respectively (120 and 32 ppm).

^{1}H Inversion recovery experiments and ^{15}N relaxation measurements

^{1}H 1D IR

1D ^{1}H IR spectra were recorded on the 900 MHz spectrometer. The selective inversion of the amide protons region was achieved with a pulse of 1200 µs length and Reburp.1000 shape centered at 8.2 ppm. A rectangular z gradient (1%) was applied during the inversion recovery delay (d7). Ten spectra were recorded with recovery delays of 20 ms, 100 ms, 150 ms, 180 ms, 200 ms, 300 ms, 400 ms, 600 ms, 1.2 s and 3 s. The spectra were recorded with the ^{1}H carrier frequency set to 4.7 ppm, 4 s relaxation delay and 32 scans.

^{1}H-^{15}N 2D IR HSQC

The 2D IR HSQC spectra was recorded on the 900 MHz spectrometer. The experiments were carried out with a relaxation delay of 4 s, 8 scans (2304 x 512 points) and the ^{1}H carrier frequency set at 4.7 ppm. Recovery delays from 100 ms to 4s were tested. The selective inversion of the amide protons region was achieved with a pulse of 1200 µs length and Reburp shape centered at 8.1 ppm. At 1200 MHz and at 700 MHz the length of this pulse was 900 µs and 1400 µs, respectively. A rectangular z gradient (1%) was applied during the inversion recovery delay.

The longitudinal relaxation rates of amide protons were measured by acquiring a series of 2D IR HSQC with the following recovery delays: 20 ms, 50 ms, 75 ms, 100 ms, 150 ms, 200 ms, 300 ms, 500 ms, 800 ms, 1.5 s and 4s. These spectra were recorded at the 700 MHz spectrometer, with a relaxation delay of 4s and 8 scans and 2048 x 512 points. R_1 rates were calculated by fitting the intensities of the peaks with the following equation $I = I_0(1 - 2e^{-R_1 t})$.

^{15}N rates and heteronuclear NOE

R_1 rates were measured at the 700 MHz spectrometer 2 by performing pseudo-3D ^{1}H-^{15}N correlation experiments in which the third "dimension" is used to store the 2D HN maps acquired with different effective relaxation delays. The spectra were acquired with 8 scans (2024 x 180 points), with a relaxation delay of 3 s and the following delays for the pseudo third dimension: 20 ms, 100 ms, 200 ms, 400 ms, 1 s, 1.5 s and 2 s. R_1 rates were calculated by fitting the intensities of the peaks with the following equation $I = I_0 e^{(-R_1 \cdot t)}$

R_2 rates were measured by performing pseudo-3D ^{1}H-^{15}N experiments at the same instrument used for R_1 measurement. The spectra were acquired with 8 scans (2024 x 180 points), with a relaxation delay of 3 s and the following delays for the pseudo third dimension: 21.4 ms, 42.8 ms, 64.2 ms, 85.6 ms, 107 ms, 149.5 ms, 235.4 ms, 299.6 ms and 428 ms. R_2 rates were calculated by fitting the intensities of the peaks with the following equation $I = I_0 e^{(-R_2 \cdot t)}$

The NOE was measured by performing a 2D ^{1}H-^{15}N experiment on the same instrument used for R_1 measurement. The spectrum was acquired with 96 scans (2024 x 180 points) and a relaxation delay of 6 s. The spectrum was repeated with a saturation block composed of a 120° hard pulse and a 5 ms delay repeated 1196 times. The NOE values were obtained for each peak by dividing the intensity of the peak in the reference spectrum by that of the same peak in the spectrum with the saturation block.

These three experiments were repeated on the same instrument also at 283 K.

CLEANEX

The CLEANEX experiment was acquired at the 900 MHz spectrometer. The spectra were acquired with 16 scans, 2048 x 512 points and a relaxation delay of 1 s. The following delays were used: 10 ms, 20 ms, 50 ms and 100 ms.

Temperature dependence experiments

The temperature dependence coefficient of amide protons' chemical shift was measured by acquiring a series of 2D ^{1}H-^{15}N HSQC on the 1200 MHz spectrometer at the following temperatures: 278 K, 285 K, 292 K and 298 K. All the experiments were acquired with 8 scans (3072 x 1024 points) and a relaxation delay of 2 s.

Data processing and analysis

All the spectra were acquired and processed using TopSpin 4.0 or above; the spectra were analyzed using TopSpin 4.0 or above and XEASY[26]. The signal to noise ratio was determined by dividing the peak height by twice the noise standard deviation, that was measured by taking a window of 0.3 ppm on the direct dimension.

c. *In silico* calculations

HydroNMR

The correlation time of TAZ2 domain in isolation was carried out with HYDRONMR[27] using the PDB structure of the protein (PDB code: 1f81[28]). Six values of bead radius from 1.5 to 3 Å were used. Standard values were used for the other variables. The values obtained refer to a temperature of 298 K and a static magnetic field of 16.4 T.

IDPConformerGenerator[29]

The starting culled database was obtained from PISCES[30] with the following constraints:

Resolution: 1.0 - 2.5

R-factor: 0.25
Sequence length: 100-800
Sequence % identity: <= 25%
X-rays entries and NMR entries included
Chains with chain break excluded
Chains with disorder included
The database generated contained 9338 structures. Loops and helices were sampled, and 5000 conformers were calculated using the build algorithm. The ldrs algorithm[31] was used to model the ID4 chain as C-terminal tail of the folded TAZ2 (PDB code: 1f81), to generate the TAZ4 conformers.

AlphaFold3[32]

AlphaFold Server web-service was used for both TAZ4 structure modelling and the prediction of the interaction between TAZ4 and ID4. Standard parameters were used.

2. Supplementary figures

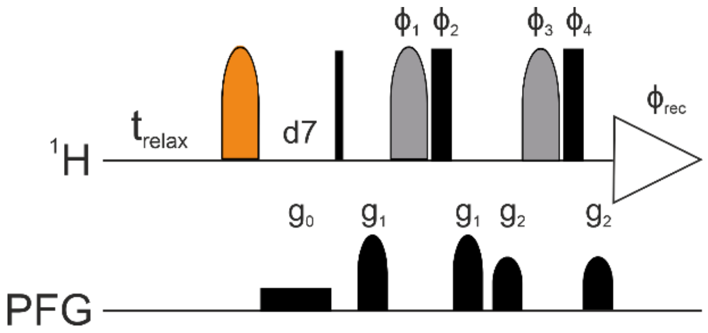

Figure S1. Pulse sequence of the ^{1}H 1D IR experiment. The orange shape represents the 180° rotation pulse selective on the amide protons. This pulse has a Reburp shape, and its duration depends on the bandwidth to cover which in turns depends on the static magnetic field. Further details are given in the methods section. d7 is the recovery delay after inversion, during which a rectangular gradient (g_0) with strength of 1% is applied. This gradient is crucial to avoid water interference. Thin black rectangles represent hard 90° rotation pulses, broad black rectangles represent hard 180° rotation pulses, grey shapes represent selective pulses on water. Black shapes represent smoothed square gradients of the durations of 1 ms. The triangle on the ^{1}H channel represents the acquisition. The pulse sequence after the d7 delay is the 1D ^{1}H excitation sculpting experiment[33]. ϕ_1 = x, y; ϕ_2 = -x, -y; ϕ_3 = x, x, y, y; ϕ_4 = -x, -x, -y, -y. ϕ_{rec} = x, -x, -x, x. g_1 = 31% g_2 = 11%. The pulse sequence script for Bruker instruments is reported in section 4 of the supplementary material.

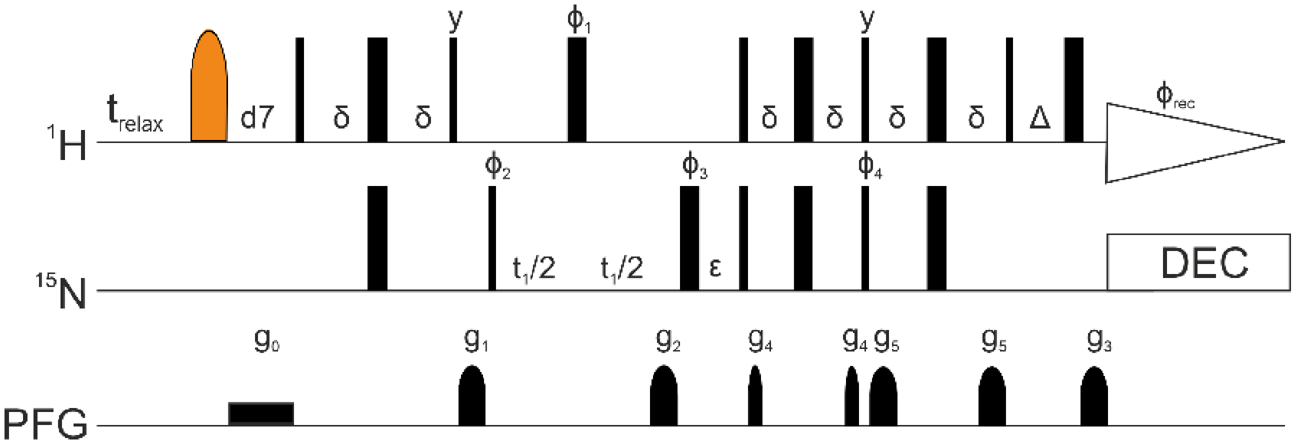

Figure S2. Pulse sequence of the ^{1}H-^{15}N 2D IR HSQC. The orange shape represents the 180° rotation pulse selective on the amide protons. This pulse has a Reburp shape, and its duration depends on the bandwidth to cover which in turns depends on the static magnetic field. Further details are given in the methods section. d7 is the recovery delay after inversion, during which a rectangular gradient (g_0) with strength of 1% is applied. This gradient is crucial to avoid water interference. Thin black rectangles represent hard 90° rotation pulses, broad black rectangles represent hard 180° rotation pulses. Black thin shapes represent smoothed square gradients of the duration of 0.5 ms (p_{19}). Black broad shapes represent smoothed square gradients of the duration of 1 ms (p_{16}). The triangle on the ^{1}H channel represents the acquisition. The rectangle on the ^{15}N channel represents the decoupling scheme. The pulse sequence after the d7 delay is the 2D HSQC with sensitivity improvement experiment[34]. ϕ_1 = x, x, -x, -x; ϕ_2 = x, -x; ϕ_3 = x, x, -x, -x; ϕ_4 = y, y, -y, -y; ϕ_{rec} = x, -x, -x, x. g_1 = 50%, g_2 = 80%, g_3 = 8.1%, g_4 = 5%, g_5 = -2%. $\delta = 1/4J_{NH}$ = 2.72 ms, $\varepsilon = t_1(0) + p_{16} + d_{16} + p_2$ = 1.23 ms, Δ = 1.21 ms. d_{16} is the delay for gradient recovery, p_2 is the length of the hard 180° rotation pulse on ^{1}H channel. The pulse sequence script for Bruker instruments is reported in section 4 of the supplementary material.

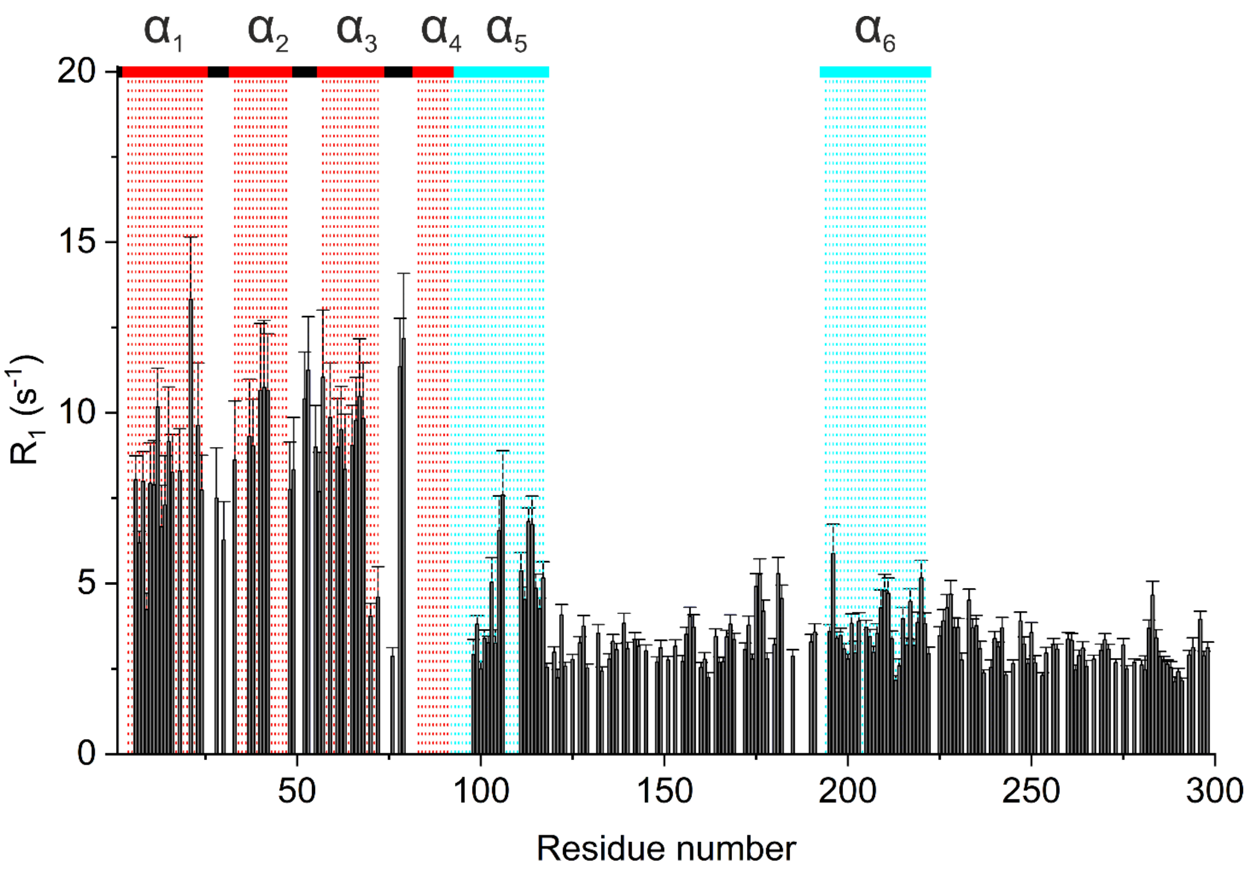

Figure S3. A series of 2D IR HN spectra acquired with increasing recovery delays were used to estimate the apparent $^{1}H^{N}$ R_1 using a mono-exponential fitting function[35]. The results of this fitting are reported in the figure as a function of the residue number.

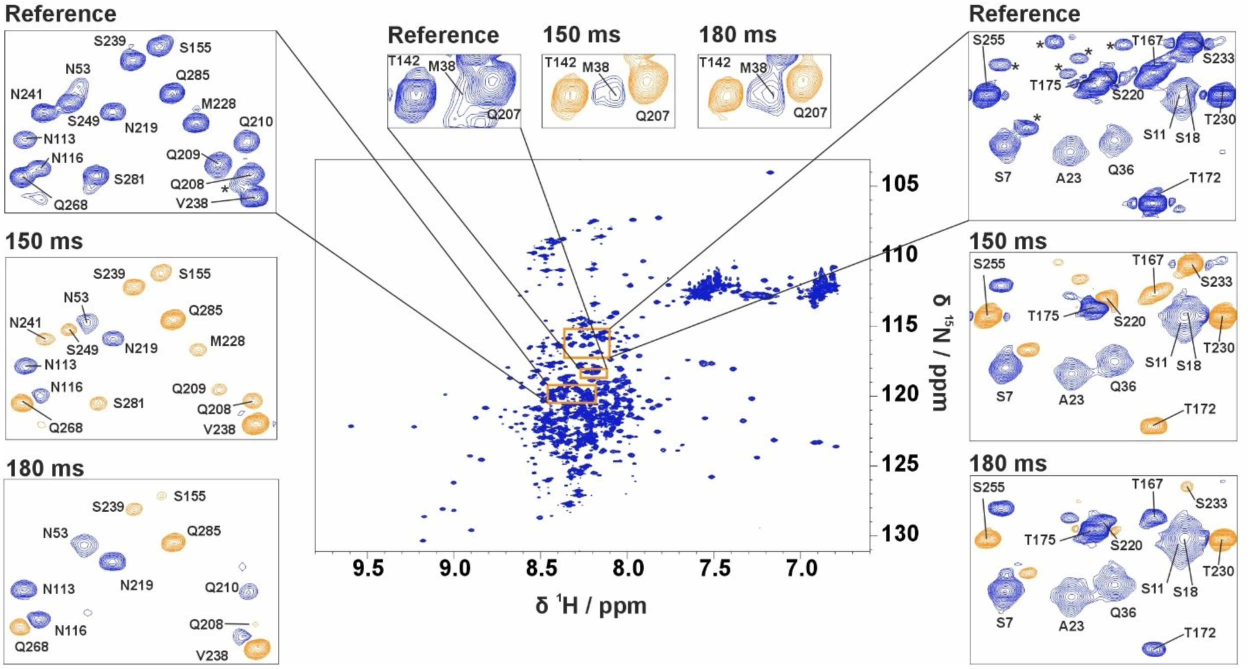

Figure S4. A 2D ^1H-^{15}N HSQC spectrum of TAZ4 recorded at 298 K and 21.1 T (900 MHz). Three regions were selected to compare the HSQC spectra obtained with and without the longitudinal enhancement relaxation filter. The boxes show enlarged portions of the 2D ^1H-^{15}N HSQC spectrum and the corresponding regions of the 2D ^1H-^{15}N IR HSQC spectra recorded with a delay (d7) of 150 ms and 180 ms, respectively. Labels indicate the assignment of the cross peaks. Asterisks indicate signals due to a minor form of ID4. Positive cross peaks are depicted with blue contours, while negative cross peaks are shown with orange contours. The contour levels of the spectra have been chosen to maximize the number of visible peaks, with the base contour level set just above the noise. All the spectra were acquired with a relaxation delay of 4 s and 8 scans. The durations of the experiments were 4 hours and 47 minutes for the 2D ^1H-^{15}N HSQC, 4 hours and 57 minutes for the 2D ^1H-^{15}N IR HSQC (d7 = 150 ms) and 5 hours for the 2D ^1H-^{15}N IR HSQC (d7 = 180 ms). Nevertheless, there is a decrease in sensitivity due to the insertion of the IR filter. As an example, the signal to noise ratio (S/N) of two representative peaks, one from the globular (Q36) and one from the disordered region (S255), estimated in the very same experiments, is reported. The S/N for Q36 is 43.5, 16.1 and 21.9, respectively, and for S255 is 622.1, 126.2 and 51.5, respectively.

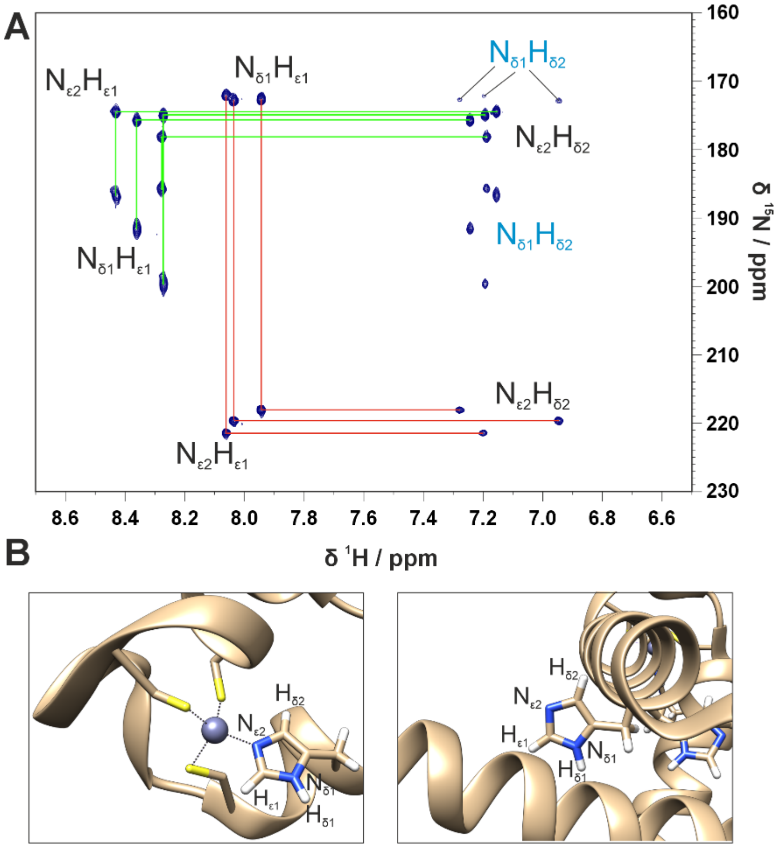

Figure S5. A) Long range 2D $^1H^{15}N$-HSQC experiment on histidine side chains recorded on a 300 μM ^{15}N TAZ4 sample (25 mM sodium acetate buffer, 50 mM sodium chloride, 0.1 mM zinc chloride, 2 mM TCEP, 0.05% sodium azide, 10% deuterium oxide) at pH 5.5 at 298 K and 21.1 T (900 MHz). The correlation pattern among the peaks of each histidine is shown in the spectrum. The solid lines identify the $^2J_{HN}$ correlations. Green and red lines indicate free and Zn(II)-bound histidine residues, respectively. Light blue labels indicate the $^3J_{HN}$ correlations. B) From left to right the two panels depict the side chain of a Zn(II)-bound histidine and a free histidine respectively (PDB 1F81).

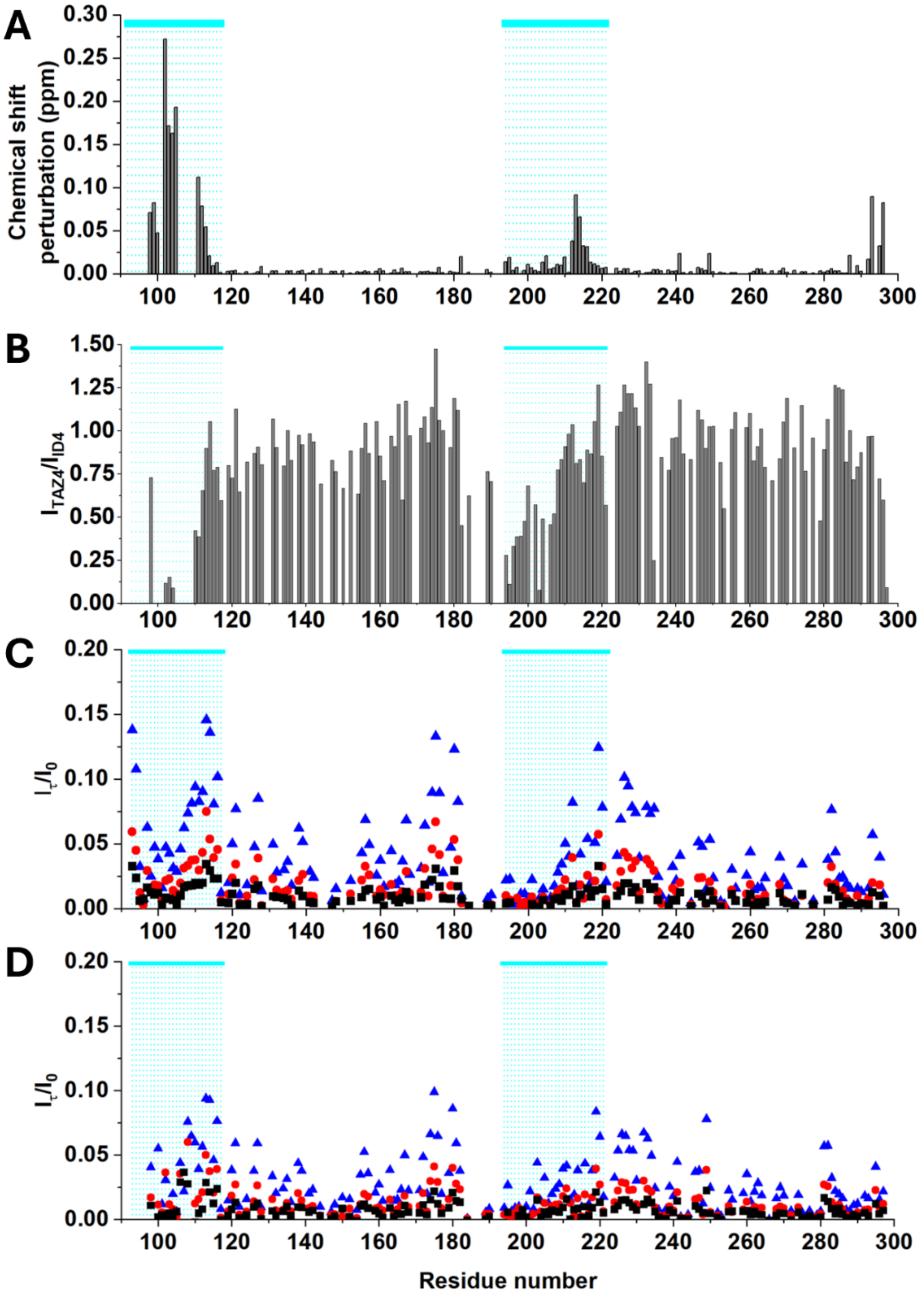

Figure S6. A) Cumulative ^{1}H and ^{15}N chemical shift perturbations obtained by comparing 2D HN spectra of ID4 in isolation and the ID4 region in the TAZ4 construct. Spectra were recorded at 298 K, pH 5.5 and 16.4 T (700 MHz). B) Intensity ratios of 2D HN cross peaks of TAZ4 and ID4. The spectra were acquired at pH 5.5, 298 K and 16.4 T (700 MHz). C) Ratios of peak intensities between CLEANEX spectra and the reference spectrum plotted against the residue number for ID4 at 298 K and 21.1 T (900 MHz). The ratios are reported for the following delays: 10 ms (black squares), 20 ms (red circles) and 50 ms (blue triangles). D) Ratios of peak intensities between CLEANEX spectra and the reference spectrum plotted against the residue number for the ID4 region in the TAZ4 construct at 298 K and 21.1 T (900 MHz). The ratios are reported for the following delays: 10 ms (black squares), 20 ms (red circles) and 50 ms (blue triangles). Cyan boxes and drop lines indicate the location of the two partially folded α-helices.

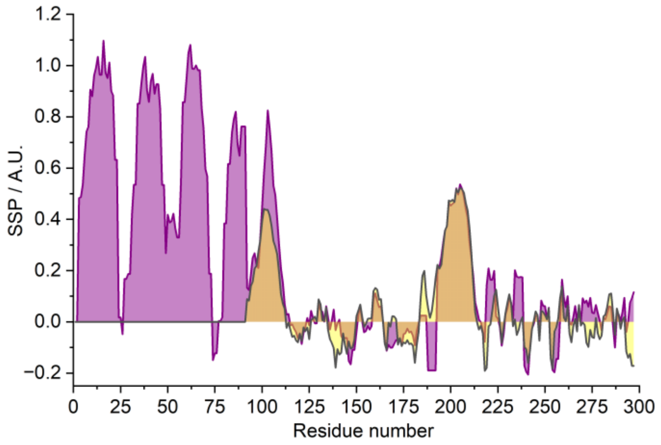

Figure S7. Secondary structure propensity of TAZ4 (purple) and isolated ID4 (yellow) calculated from the chemical shifts at 298 K and 16.4 T (700 MHz). Positive and negative values indicate propensity to form α-helices and β-strands respectively. The calculation was done with the ncIDP online tool[36]**.**

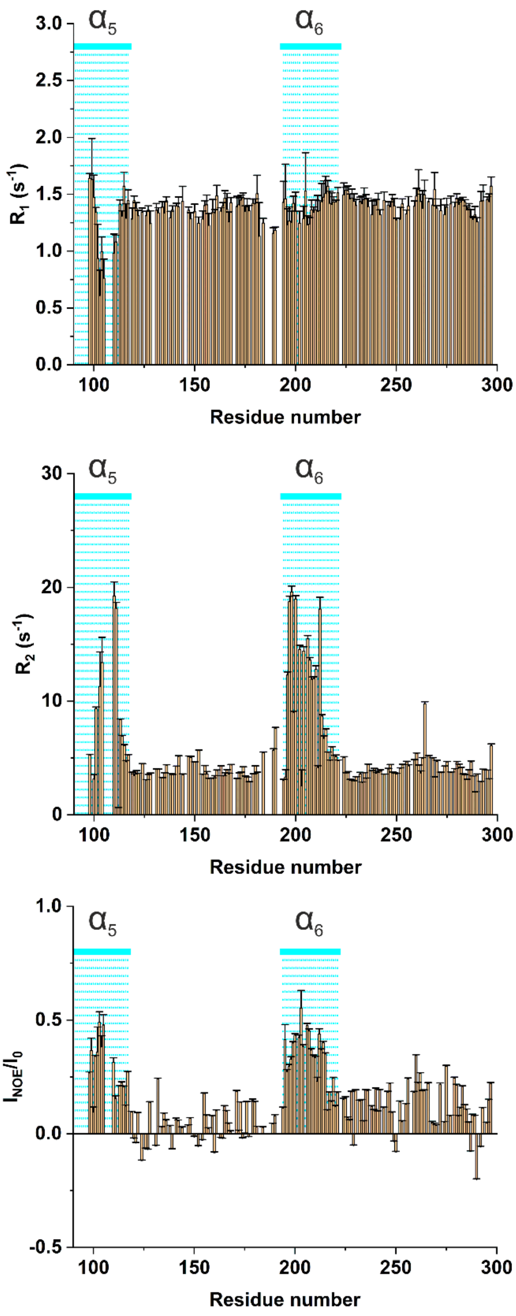

Figure S8. ^{15}N relaxation properties of the ID4 region within the TAZ4 construct measured at 283 K and 16.4 T (700 MHz).

```
P300               1 ATQSPGDSRRLSIQRAIQSLVHAAQCRNANCSLPSCQKMKRVVQHTKGCK     50
                      ..||.:|||||||||||||||||||||||||||||||||||||||||||
TAZ4               1 -MGSPQESRRLSIQRAIQSLVHAAQCRNANCSLPSCQKMKRVVQHTKGCK     49

P300              51 RKTNGGCPICKQLIALAAYHAKHCQENKCPVPFCLNIKQKLRQQQLQHRL    100
                     ||||||||:||||||||||||||||||||||||||||||.||||||:||||
TAZ4              50 RKTNGGCPVCKQLIALAAYHAKHCQENKCPVPFCLNIKHKLRQQQIQHRL     99

P300             101 QQAQMLRRRMASMQ    114
                     ||||::|||||:|.
TAZ4             100 QQAQLMRRRMATMN    113
```

Figure S9. Pairwise sequence alignment between the TAZ2 domain of P300 and the corresponding portion of the TAZ4 construct. The alignment was carried out with the EMBOSS Needle online tool[37].

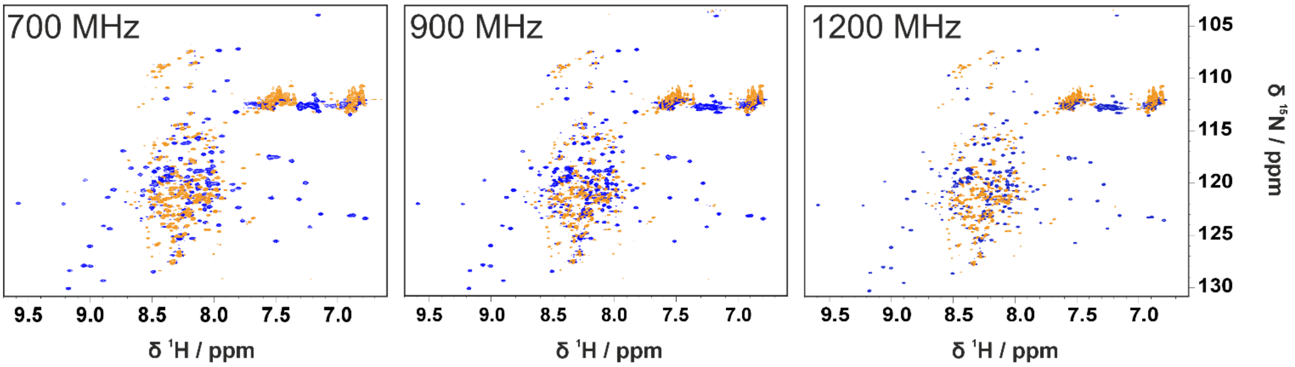

Figure S10. 2D ^{1}H-^{15}N IR HSQC spectra of TAZ4 recorded at 298 K using spectrometers with magnetic field strengths of 16.4, 21.1, and 28.2 T (700, 900, and 1200 MHz) with a relaxation delay (d7) of 150 ms. The difference in magnetic field strength does not significantly affect the discrimination capability of the longitudinal enhancement relaxation filter. Positive and negative cross peaks are represented by blue and orange contours respectively.

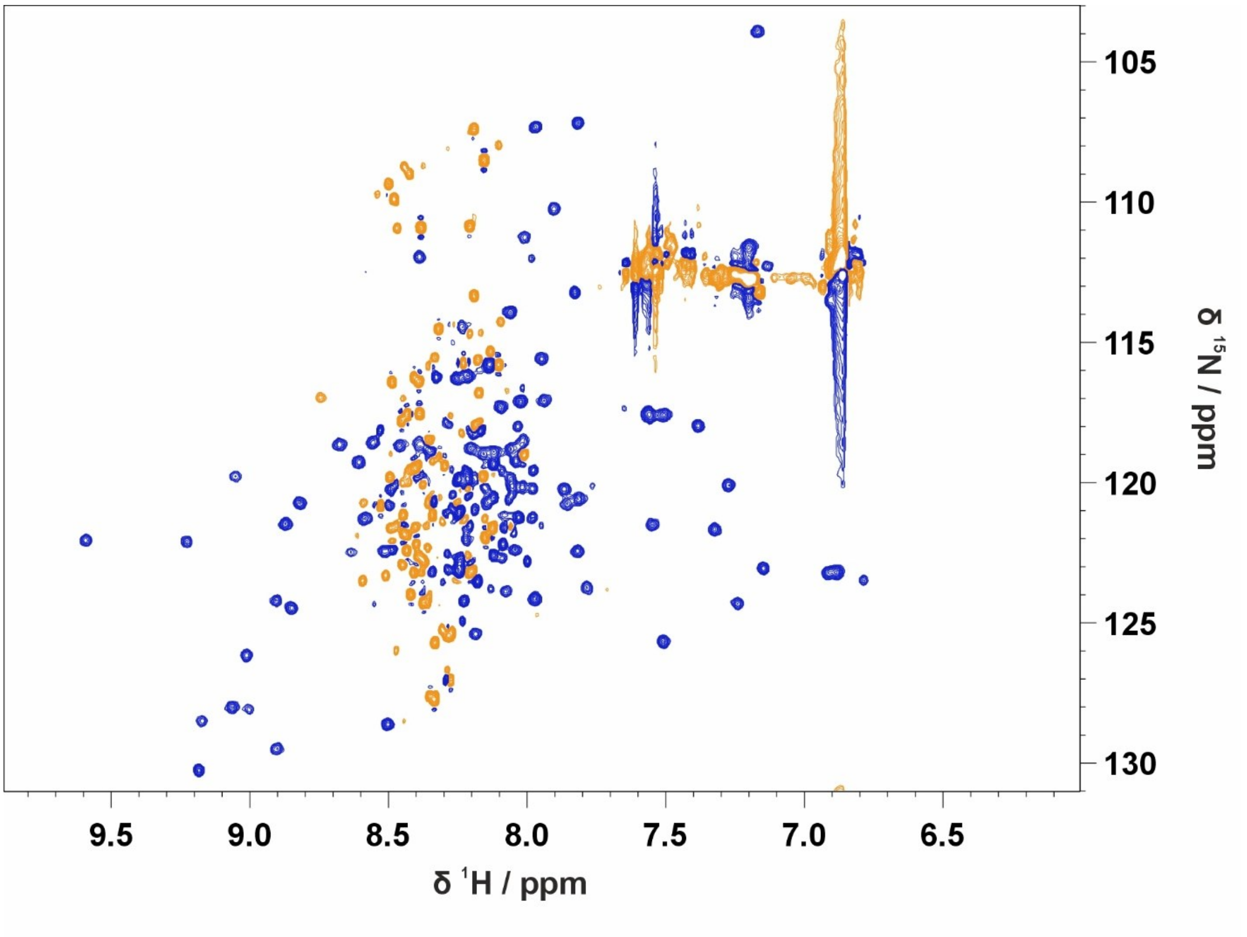

Figure S11. 2D ^{1}H-^{15}N heteronuclear NOE spectrum recorded on TAZ4 at 298 K at 16.4 T (700 MHz). This experiment allows to discriminate between regions of proteins characterized by very different local dynamics. Positive and negative cross peaks are represented by blue and orange contours respectively. The total duration of the experiment was 2 days and 11 hours.

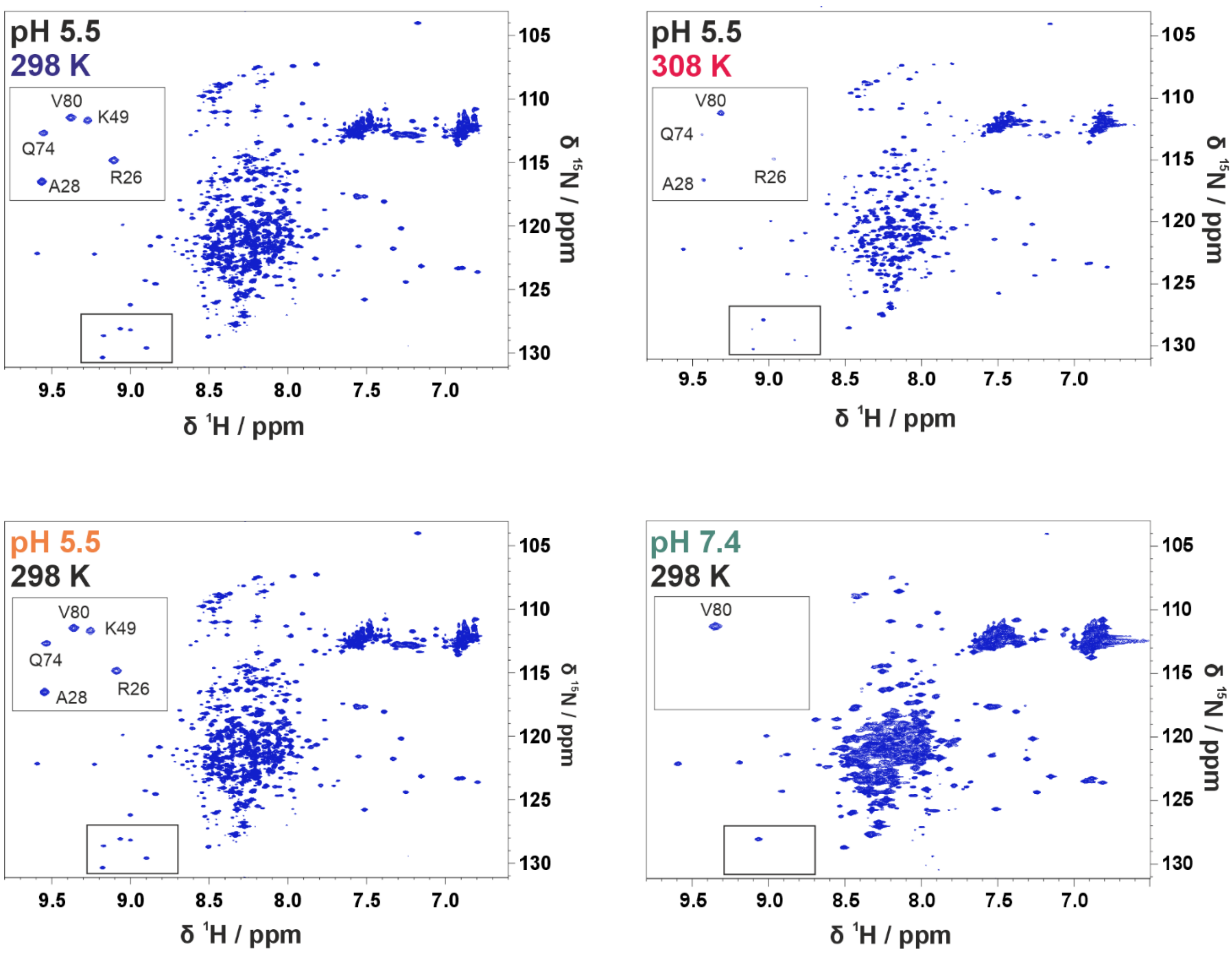

Figure S12. Effect of temperature and pH on the ¹H-¹⁵N HSQC spectrum of TAZ4. The spectrum at pH 7.4 was acquired in 25 mM potassium phosphate buffer, while those at pH 5.5 were acquired in 25 mM sodium acetate buffer. The insets show the same region of the spectrum that include peaks of the TAZ2 domain. A significant reduction of the number of signals due to the disordered region of the protein is present, which does not completely resolve the resonances of the globular domain. There is an overall broadening of the lines and the disappearance of several cross peaks, including those of the more exposed residues in the globular domain. The contour levels of the spectra have been chosen to maximize the number of visible peaks, with the base contour level set just above the noise.

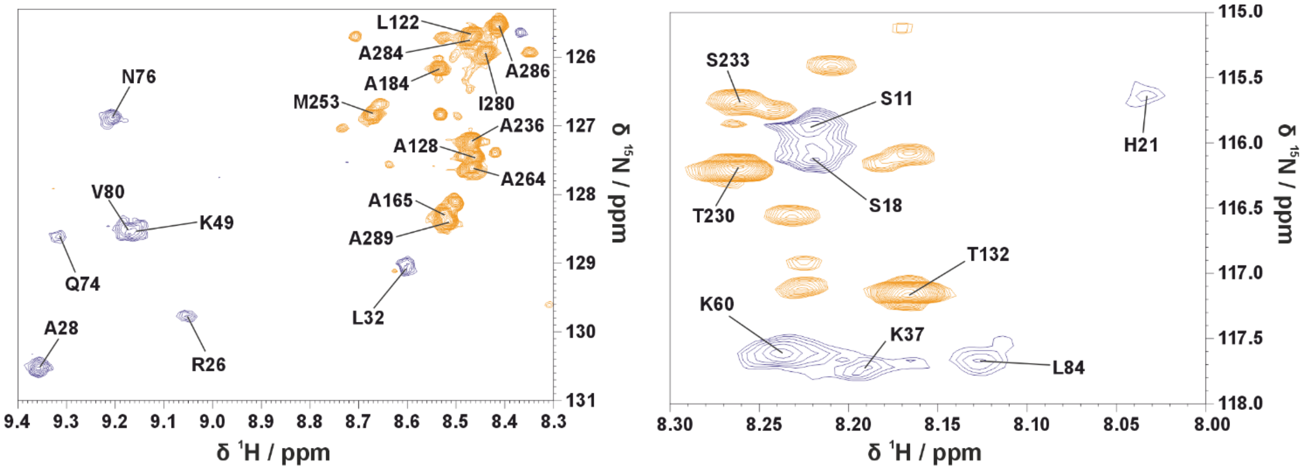

Figure S13. Temperature and pH conditions can be adjusted for the application of the T_1 filtering strategy proposed in this work. For instance, these panels show two regions of the ^{1}H-^{15}N 2D IR HSQC spectrum of a TAZ4 sample at pH 6.5 recorded at 283 K on a 21.1 T (900 MHz) spectrometer, acquired with a recovery delay of 120 ms. Positive cross peaks are depicted with blue contours, while negative cross peaks are shown with orange contours. In these conditions, exchange with solvent is still moderate and it is possible to select the cross peaks deriving from the globular domain from those deriving from the disordered region.

3. Pulse sequences

^{1}H 1D IR experiment

```
;IR_zgesgp
;IR_1D sequence
;water suppression using excitation sculpting with gradients
;with selective inversion recovery scheme
;T.-L. Hwang & A.J. Shaka, J. Magn. Reson.,
;   Series A 112 275-279 (1995)
;
;$CLASS=HighRes
;$DIM=1D
;$TYPE=
;$SUBTYPE=
;$COMMENT=

prosol relations=<triple>

#include <Avance.incl>
#include <Grad.incl>
#include <Delay.incl>

"p2=p1*2"
"d12=20u"

"TAU=de+p1*2/3.1416+50u"

"acqt0=0"
;baseopt_echo

1 ze
2 30m
  d12 BLKGRAD
  d1
  50u UNBLKGRAD
  d12 pl0:f1
  (p14:sp14 ph1):f1
  d7 gron0
  4u groff
  d12 pl1:f1
  p1 ph1
  p16:gp1
  d16 pl0:f1
  (p12:sp1 ph2:r):f1
  4u
  d12 pl1:f1
  p2 ph3
  4u
  p16:gp1
  d16
  TAU
  p16:gp2
  d16 pl0:f1
  (p12:sp1 ph4:r):f1
  4u
  d12 pl1:f1
  p2 ph5

  4u
  p16:gp2
  d16
  go=2 ph31
  30m mc #0 to 2 F0(zd)
  4u BLKGRAD
exit
```

```
ph1=0
ph2=0 1
ph3=2 3
ph4=0 0 1 1
ph5=2 2 3 3
ph31=0 2 2 0

;pl0 : 0W
;pl1 : f1 channel - power level for pulse (default)
;sp1 : f1 channel - shaped pulse 180 degree
;sp14: f1 channel - shaped pulse 180 degree (Reburp.1000)
;p1 : f1 channel -  90 degree high power pulse
;p2 : f1 channel - 180 degree high power pulse
;p12: f1 channel - 180 degree shaped pulse (Squa100.1000)   [2 msec]
;p14: f1 channel - 180 degree shaped pulse for inversion recovery
;p16: homospoil/gradient pulse
;d1 : relaxation delay; 1-5 * T1
;d7 : recovery delay after inversion
;d12: delay for power switching                    [20 usec]
;d16: delay for homospoil/gradient recovery
;ns: 8 * n, total number of scans: NS * TD0
;ds: 4


;use gradient ratio:    gp 1 : gp 2
;                        31 :   11

;for z-only gradients:
;gpz0: 1%
;gpz1: 31%
;gpz2: 11%

;use gradient files:
;gpnam1: SMSQ10.100
;gpnam2: SMSQ10.100
;$Id:$
```

^{1}H-^{15}N 2D IR HSQC experiment

```
;IR_hsqcetf3gpsi2
;HSQC
;IR_2D H-1/X correlation via double inept transfer
;   using sensitivity improvement
;phase sensitive using Echo/Antiecho-TPPI gradient selection
;with decoupling during acquisition
;with selective inversion recovery scheme
;using trim pulses in inept transfer
;using f3 - channel
;with gradients in back-inept
;
;A.G. Palmer III, J. Cavanagh, P.E. Wright & M. Rance, J. Magn.
;   Reson. 93, 151-170 (1991)
;L.E. Kay, P. Keifer & T. Saarinen, J. Am. Chem. Soc. 114,
;   10663-5 (1992)
;J. Schleucher, M. Schwendinger, M. Sattler, P. Schmidt, O. Schedletzky,
;   S.J. Glaser, O.W. Sorensen & C. Griesinger, J. Biomol. NMR 4,
;   301-306 (1994)
;
;$CLASS=HighRes
;$DIM=2D
;$TYPE=
;$SUBTYPE=
;$COMMENT=

#include <Avance.incl>
#include <Grad.incl>
#include <Delay.incl>

"p2=p1*2"
"p22=p21*2"
"d11=30m"
"d12=20u"
"d26=1s/(cnst4*4)"
"d0=3u"
"in0=inf1/2"


"DELTA1=p16+d16-p1*0.78+de+8u"
"DELTA2=d24-p19-d16"
"DELTA3=d26-p16-d16"

#   ifdef LABEL_CN
"DELTA=p16+d16+larger(p2,p14)+d0*2"
#   else
"DELTA=p16+d16+p2+d0*2"
#   endif /*LABEL_CN*/

"acqt0=0"
baseopt_echo


1 ze
  d11 pl16:f3
2 d1 do:f3
3 50u UNBLKGRAD
  d12 pl0:f1
  (p31:sp31 ph1):f1
    d7 gron0
  4u groff
  d12 pl1:f1
  (p1 ph1)
  d26 pl3:f3
  (center (p2 ph1) (p22 ph6):f3 )
  d26
#   ifdef TRIMP
```

```
 p28 ph1
#   endif /* TRIMP */
 (p1 ph2)
 3u
 p16:gp1
 d16
 (p21 ph3):f3
 d0
#   ifdef LABEL_CN
 (center (p2 ph7) (p14:sp3 ph1):f2 )
#   else
 (p2 ph7)
#   endif /*LABEL_CN*/
 d0
 p16:gp2*EA
 d16
 (p22 ph4):f3
 DELTA
 (center (p1 ph1) (p21 ph4):f3 )
 p19:gp4
 d16
 DELTA2
 (center (p2 ph1) (p22 ph1):f3 )
 DELTA2
 p19:gp4
 d16
 (center (p1 ph2) (p21 ph5):f3 )
 p16:gp5
 d16
 DELTA3
 (center (p2 ph1) (p22 ph1):f3 )
 DELTA3
 p16:gp5
 d16
 (p1 ph1)
 DELTA1
 (p2 ph1)
 4u
 p16:gp3
 d16 pl16:f3
 4u BLKGRAD
 go=2 ph31 cpd3:f3
 d1 do:f3 mc #0 to 2
   F1EA(calgrad(EA) & calph(ph5, +180), caldel(d0, +in0) & calph(ph3, +180) & calph(ph6, +180) & calph(ph31, +180))
exit

ph1=0
ph2=1
ph3=0 2
ph4=0 0 2 2
ph5=1 1 3 3
ph6=0
ph7=0 0 2 2
ph31=0 2 2 0


;pl0 : 0W
;pl1 : f1 channel - power level for pulse (default)
;pl3 : f3 channel - power level for pulse (default)
;pl16: f3 channel - power level for CPD/BB decoupling
;sp3: f2 channel - shaped pulse 180 degree (adiabatic)
;sp31: f1 channel - shaped pulse 180 degree (Reburp.1000)
;p1 : f1 channel -  90 degree high power pulse
;p2 : f1 channel - 180 degree high power pulse
;p14: f2 channel - 180 degree shaped pulse for inversion (adiabatic)
;p16: homospoil/gradient pulse                    [1 msec]
;p19: gradient pulse 2                            [500 usec]
```

```
;p21: f3 channel -  90 degree high power pulse
;p22: f3 channel - 180 degree high power pulse
;p28: f1 channel - trim pulse                     [1 msec]
;p31: f1 channel - 180 degree shaped pulse for inversion recovery
;d0 : incremented delay (2D)                      [3 usec]
;d1 : relaxation delay; 1-5 * T1
;d7 : recovery delay after inversion
;d11: delay for disk I/O                          [30 msec]
;d12: delay for power switching
;d16: delay for homospoil/gradient recovery
;d24: 1/(4J)YH for YH
;     1/(8J)YH for all multiplicities
;d26: 1/(4J(YH))
;cnst4: = J(YH)
;inf1: 1/SW(X) = 2 * DW(X)
;in0: 1/(2 * SW(X)) = DW(X)
;nd0: 2
;ns: 1 * n
;ds: >= 16
;td1: number of experiments
;FnMODE: echo-antiecho
;cpd3: decoupling according to sequence defined by cpdprg3
;pcpd3: f3 channel - 90 degree pulse for decoupling sequence


;for z-only gradients:
;gpz0: 1%
;gpz1: 50%
;gpz2: 80%
;gpz3: 8.1% for N-15, 20.1% for C-13
;gpz4: 5%
;gpz5: -2%

;use gradient files:
;gpnam1: SMSQ10.100
;gpnam2: SMSQ10.100
;gpnam3: SMSQ10.100
;gpnam4: SMSQ10.100
;gpnam5: SMSQ10.100


                                  ;preprocessor-flags-start
;LABEL_CN: for C-13 and N-15 labeled samples start experiment with
;             option -DLABEL_CN (eda: ZGOPTNS)
;TRIMP: to use trimpulse p28@pl1 start experiment with
;          option -DTRIMP (eda: ZGOPTNS)
                                  ;preprocessor-flags-end



;$Id:$
```

3D IR HNCO

```
;b_hncogp3d
;IR_best-HNCO
;3D sequence with
;   inverse correlation for triple resonance using multiple
;      inept transfer steps
;
;      F1(H) -> F3(N) -> F2(C=O,t1) -> F3(N,t2) -> F1(H,t3)
;
;on/off resonance Ca and C=O pulses using shaped pulse
;using shaped pulses for inversion and refocussing on f3
;phase sensitive (t1)
;phase sensitive using Echo/Antiecho (t2)
;using semi constant time in t2
;with selective inversion recovery scheme
;
;P. Schanda, H. v. Melckebeke & B. Brutscher,
;   J. Am. Chem. Soc. 128, 9042-9043 (2006)
;E. Lescop, P. Schanda & B. Brutscher,
;   J. Magn. Reson.  187 163-169 (2007)
;(S. Grzesiek & A. Bax, J. Magn. Reson. 96, 432 - 440 (1992))
;(J. Schleucher, M. Sattler & C. Griesinger,
;   Angew. Chem. Int. Ed. 32, 1489-1491 (1993))
;(L.E. Kay, G.Y. Xu & T. Yamazaki, J. Magn. Reson. A109,
;   129-133 (1994))
;
;$CLASS=HighRes
;$DIM=3D
;$TYPE=
;$SUBTYPE=
;$COMMENT=

prosol relations=<triple>

#include <Avance.incl>
#include <Grad.incl>
#include <Delay.incl>

"d11=30m"

"d23=14.5m"
"d26=2.7m"

"p29=250u"

#   ifdef CALC_SP
"p41=(bwfac25/(cnst55*cnst51*bf1))*1000000"
"spw25=plw1/((p41*90.0)/(p1*totrot25))*((p41*90.0)/(p1*totrot25))*(integfac25*integfac25)"
"spw27=plw1/((p41*90.0)/(p1*totrot27))*((p41*90.0)/(p1*totrot27))*(integfac27*integfac27)"
"spoal25=1"
"spoal27=0"
"p42=(bwfac26/(cnst55*cnst52*bf1))*1000000"
"spw26=plw1/((p42*90.0)/(p1*totrot26))*((p42*90.0)/(p1*totrot26))*(integfac26*integfac26)"
"spoal26=0.5"
"p43=(bwfac28/(cnst55*cnst53*bf1))*1000000"
"spw28=plw1/((p43*90.0)/(p1*totrot28))*((p43*90.0)/(p1*totrot28))*(integfac28*integfac28)"
"spw29=plw1/((p43*90.0)/(p1*totrot29))*((p43*90.0)/(p1*totrot29))*(integfac29*integfac29)"
"spoal28=1"
"spoal29=0"
#   endif /*CALC_SP*/

"d0=3u"
"d10=3u"
"d12=20u"
"d29=3u"
```

```
"d30=d23-p43-4u-p21*4/PI"

"in0=inf1/2"
"in10=inf2/2"

"FACTOR2=d30*10000000*2/td2"
"INCR2=FACTOR2/10000000"

"if ( INCR2 > in10 ) { in30 = in10; } else { in30 = INCR2; }"
"if ( INCR2 > in10 ) { in29 = 0; } else { in29=in10-INCR2; }"

"TAU=larger(p14,p44)"

"DELTA=d0*2+larger(TAU,p56)-p14"
"DELTA1=d26-p29-d16-p41*cnst41-larger(p42,p56)/2"
"DELTA2=d23-d26-p44-p16-d16-p14-d29"
"DELTA3=d26-p19-d16-p42/2"
"DELTA4=d26-p29-d16-p43*cnst43-larger(p42,p56)/2"
"DELTA5=p16+d16+de+8u"
"DELTA6=d23-larger(p42,p57)/2"
"DELTA7=d23-larger(p42,p57)/2-p44-d26"
"DELTA8=d26-p14-d10"

"spoff2=0"
"spoff3=0"
"spoff5=bf2*(cnst22/1000000)-o2"
"spoff8=0"

"spoff25=bf1*(cnst54/1000000)-o1"
"spoff26=bf1*(cnst54/1000000)-o1"
"spoff27=bf1*(cnst54/1000000)-o1"
"spoff28=bf1*(cnst54/1000000)-o1"
"spoff29=bf1*(cnst54/1000000)-o1"
"spoff30=0"

aqseq 321

"acqt0=0"
baseopt_echo


1 d11 ze
  d11 pl26:f3
2 d11 do:f3
3 d1
  50u UNBLKGRAD
  d12 pl0:f1
  (p31:sp31 ph1):f1
    d7 gron0
  4u groff
  d12 pl1:f1
  (p41:sp25 ph1)
  p29:gp3
  d16
  DELTA1
  (center (p42:sp26 ph1) (p56:sp39 ph1):f3 )
  DELTA1
  p29:gp3
  d16
  (p41:sp27 ph2):f1
  p16:gp4
  d16 pl3:f3
  (p21 ph3):f3
  DELTA6
  (center (p14:sp3 ph1):f2 (p57:sp40 ph1):f3 )
  DELTA7
  (p44:sp30 ph1)
```

```
 d26 pl3:f3
 (p21 ph1):f3
 (p44:sp30 ph1)
 p16:gp5
 d16
 (p44:sp30 ph1)
 (p13:sp2 ph4):f2
 d0
 (center (p44:sp30 ph1) (p14:sp5 ph1):f2 (p56:sp39 ph7):f3 )
 d0
 4u
 (p14:sp3 ph1):f2
 DELTA
 (p14:sp5 ph1):f2
 4u
 (p13:sp8 ph1):f2
 p16:gp6
 d16 pl3:f3
 (p44:sp30 ph1)
 (p21 ph8):f3
 2u
 (p56:sp39 ph1):f3
 d10
 (p14:sp5 ph1):f2
 DELTA8
 (p44:sp30 ph1)
 DELTA2
 p16:gp1*EA
 d16
 (p14:sp3 ph1):f2
 d29
 (p56:sp39 ph7):f3
 d30
 2u pl3:f3
 (p43:sp28 ph1)
 (p21 ph5):f3
 p19:gp7
 d16
 DELTA3
 (center (p42:sp26 ph1) (p57:sp40 ph1):f3 )
 DELTA3
 p19:gp7
 d16 pl3:f3
 (p21 ph6):f3
 (p43:sp29 ph2)
 p29:gp8
 d16
 DELTA4
 (center (p42:sp26 ph1) (p56:sp39 ph1):f3 )
 DELTA4
 p29:gp8
 d16
 (p43:sp28 ph1)
 DELTA5
 (p42:sp26 ph1)
 4u
 p16:gp2
 d16 pl26:f3
 4u BLKGRAD
 go=2 ph31 cpd3:f3
 d11 do:f3 mc #0 to 2
   F1PH(calph(ph4, +90), caldel(d0, +in0))
   F2EA(calgrad(EA) & calph(ph6, +180), caldel(d10, +in10) & caldel(d29, +in29) & caldel(d30, -in30) & calph(ph8, +180) &
calph(ph31, +180))
 TAU
exit
```

```
ph1=0
ph2=1
ph3=0 0 0 0 2 2 2 2
ph4=0 2
ph5=0 0 2 2
ph6=1 1 3 3
ph7=0 0 0 0 2 2 2 2
ph8=0
ph31=0 2 2 0 2 0 0 2

;pl0 : 0W
;pl3 : f3 channel - power level for pulse (default)
;pl26: f3 channel - power level for CPD/BB low power decoupling
;sp2: f2 channel - shaped pulse  90 degree  (C=O on resonance)
;sp3: f2 channel - shaped pulse 180 degree  (C=O on resonance)
;sp5: f2 channel - shaped pulse 180 degree  (Ca off resonance)
;sp8: f2 channel - shaped pulse  90 degree  (C=O on resonance)
;              for time reversed pulse
;sp25: f1 channel - shaped pulse  90 degree (Pc9_4_90.1000)
;sp26: f1 channel - shaped pulse 180 degree (Reburp.1000)
;sp27: f1 channel - shaped pulse  90 degree (Pc9_4_90.1000)
;              for time reversed pulse
;sp28: f1 channel - shaped pulse  90 degree (Eburp2.1000)
;sp29: f1 channel - shaped pulse  90 degree (Eburp2tr.1000)
;              for time reversed pulse
;sp30: f1 channel - shaped pulse 180 degree (Bip720,50,20.1)
;sp31: f1 channel - shaped pulse 180 degree (Reburp.1000)
;sp39: f3 channel - shaped pulse 180 degree (Bip720,50,20.1)
;sp40: f3 channel - shaped pulse 180 degree (Reburp.1000)
;p13: f2 channel -  90 degree shaped pulse
;p14: f2 channel - 180 degree shaped pulse
;p16: homospoil/gradient pulse                    [1 msec]
;p19: gradient pulse 2                            [500 usec]
;p21: f3 channel -  90 degree high power pulse
;p29: gradient pulse 3                            [250 usec]
;p31: f1 channel - 180 degree shaped pulse for inversion recovery
;p41: f1 channel -  90 degree shaped pulse for excitation
;                 Pc9_4_90.1000           (2.2ms at 600.13 MHz)
;p42: f1 channel - 180 degree shaped pulse for refocussing
;                 Reburp.1000             (1.4ms at 600.13 MHz)
;p43: f1 channel -  90 degree shaped pulse for excitation
;                 Eburp2.1000/Eburp2tr.1000 (1.7ms at 600.13 MHz)
;p44: f1 channel - 180 degree shaped pulse for refocussing
;                 Bip720,50,20.1          (200us at 600.13 MHz)
;p56: f3 channel - 180 degree shaped pulse for inversion
;                 Bip720,50,20.1          (500us at 600.13 MHz)
;p57: f3 channel - 180 degree shaped pulse for refocussing
;                 Reburp.1000             (1.6ms at 600.13 MHz)
;d0 : incremented delay (F1 in 3D)                [3 usec]
;d1 : relaxation delay; 1-5 * T1
;d7 : recovery delay after inversion
;d10: incremented delay (F2 in 3D)                [3 usec]
;d11: delay for disk I/O                          [30 msec]
;d12: delay for power switching                   [20 usec]
;d16: delay for homospoil/gradient recovery
;d23: 1/(4J(NCO)                                  [14.5 msec]
;d26: 1/(4J(NH)                                   [2.7 msec]
;d29: incremented delay (F2 in 3D)                [3 usec]
;d30: decremented delay (F2 in 3D) = d23-p43-4u-p21*4/PI
;cnst21: CO chemical shift (offset, in ppm)
;cnst22: Calpha chemical shift (offset, in ppm)
;cnst41: compensation of chemical shift evolution during p41
;        Pc9_4_90.1000: 0.529
;cnst43: compensation of chemical shift evolution during p43
;        Eburp2.1000: 0.5
;cnst51: scaling factor for p41 to compensate for transition region
```

```
;	Pc9_4_90.1000: 1.172
;cnst52: scaling factor for p42 to compensate for transition region
;	Reburp.1000: 1.426
;cnst53: scaling factor for p43 to compensate for transition region
;	Eburp2.1000: 1.000
;cnst54: H(N) chemical shift (offset, in ppm)
;cnst55: H(N) bandwidth (in ppm)
;o2p: CO chemical shift (cnst21)
;inf1: 1/SW(CO) = 2 * DW(CO)
;inf2: 1/SW(N) = 2 * DW(N)
;in0: 1/(2 * SW(CO)) =  DW(CO)
;nd0: 2
;in10: 1/(2 * SW(N)) = DW(N)
;nd10: 2
;in29: = (1 - k2) * in10
;in30: = k2 * in10
;ns: 8 * n
;ds: >= 16
;aq: <= 50 msec
;td1: number of experiments in F1
;td2: number of experiments in F2
;FnMODE: States-TPPI (or TPPI) in F1
;FnMODE: echo-antiecho in F2
;cpd3: decoupling according to sequence defined by cpdprg3: garp4.p62
;pcpd3: f3 channel - 90 degree pulse for decoupling sequence
;cpdprg3: garp4.p62


;for z-only gradients:
;gpz0: 1%
;gpz1: 80%
;gpz2: 8.1%
;gpz3: 11%
;gpz4: 70%
;gpz5: 40%
;gpz6: 75%
;gpz7: 29%
;gpz8: 17%

;use gradient files:
;gpnam1: SMSQ10.100
;gpnam2: SMSQ10.100
;gpnam3: SMSQ10.32
;gpnam4: SMSQ10.100
;gpnam5: SMSQ10.100
;gpnam6: SMSQ10.100
;gpnam7: SMSQ10.50
;gpnam8: SMSQ10.32

                                        ;preprocessor-flags-start
;CALC_SP: for calculation of all bandselective Proton pulses based on cnst54 and cnst55
;	option -DCALC_SP (eda: ZGOPTNS)
                                        ;preprocessor-flags-end

;$Id:$
```

3D IR HNCA

```
;IR_b_hncagp3d
;avance-version (18/11/30)
;IR_best-HNCA
;3D sequence with
;   inverse correlation for triple resonance using multiple
;      inept transfer steps
;
;      F1(H) -> F3(N) -> F2(Ca,t1) -> F3(N,t2) -> F1(H,t3)
;
;with selective inversion recovery scheme
;on/off resonance Ca and C=O pulses using shaped pulse
;using shaped pulses for inversion and refocussing on f3
;phase sensitive (t1)
;phase sensitive using Echo/Antiecho (t2)
;using semi constant time in t2
;with selective inversion recovery scheme
;
;P. Schanda, H. v. Melckebeke & B. Brutscher,
;   J. Am. Chem. Soc. 128, 9042-9043 (2006)
;E. Lescop, P. Schanda & B. Brutscher,
;   J. Magn. Reson.  187 163-169 (2007)
;(S. Grzesiek & A. Bax, J. Magn. Reson. 96, 432 - 440 (1992))
;(J. Schleucher, M. Sattler & C. Griesinger,
;   Angew. Chem. Int. Ed. 32, 1489-1491 (1993))
;(L.E. Kay, G.Y. Xu & T. Yamazaki, J. Magn. Reson. A109,
;   129-133 (1994))
;
;$CLASS=HighRes
;$DIM=3D
;$TYPE=
;$SUBTYPE=
;$COMMENT=

prosol relations=<triple>

#include <Avance.incl>
#include <Grad.incl>
#include <Delay.incl>

"d11=30m"
"d23=12m"
"d26=2.7m"
"p29=250u"


#   ifdef CALC_SP
"p41=(bwfac25/(cnst55*cnst51*bf1))*1000000"
"spw25=plw1/((p41*90.0)/(p1*totrot25))*((p41*90.0)/(p1*totrot25))*(integfac25*integfac25)"
"spw27=plw1/((p41*90.0)/(p1*totrot27))*((p41*90.0)/(p1*totrot27))*(integfac27*integfac27)"
"spoal25=1"
"spoal27=0"

"p42=(bwfac26/(cnst55*cnst52*bf1))*1000000"
"spw26=plw1/((p42*90.0)/(p1*totrot26))*((p42*90.0)/(p1*totrot26))*(integfac26*integfac26)"
"spoal26=0.5"

"p43=(bwfac28/(cnst55*cnst53*bf1))*1000000"
"spw28=plw1/((p43*90.0)/(p1*totrot28))*((p43*90.0)/(p1*totrot28))*(integfac28*integfac28)"
"spw29=plw1/((p43*90.0)/(p1*totrot29))*((p43*90.0)/(p1*totrot29))*(integfac29*integfac29)"
"spoal28=1"
"spoal29=0"
#   endif /*CALC_SP*/


"d0=3u"
```

```
"d10=3u"
"d12=20u"
"d29=3u"
"d30=d23-p43-4u-p21*4/PI"

"in0=inf1/2"
"in10=inf2/2"

"FACTOR2=d30*10000000*2/td2"
"INCR2=FACTOR2/10000000"

"if ( INCR2 > in10 ) { in30 = in10; } else { in30 = INCR2; }"
"if ( INCR2 > in10 ) { in29 = 0; } else { in29=in10-INCR2; }"

"TAU=larger(p14,p44)"

"DELTA=d0*2+larger(TAU,p56)-p14"
"DELTA1=d26-p29-d16-p41*cnst41-larger(p42,p56)/2"
"DELTA2=d23-d26-p44-p16-d16-p14-d29"
"DELTA3=d26-p19-d16-p42/2"
"DELTA4=d26-p29-d16-p43*cnst43-larger(p42,p56)/2"
"DELTA5=p16+d16+de+8u"
"DELTA6=d23-larger(p42,p57)/2"
"DELTA7=d23-larger(p42,p57)/2-p44-d26"
"DELTA8=d26-p14-d10"

"spoff2=0"
"spoff3=0"
"spoff5=bf2*(cnst21/1000000)-o2"
"spoff8=0"
"spoff25=bf1*(cnst54/1000000)-o1"
"spoff26=bf1*(cnst54/1000000)-o1"
"spoff27=bf1*(cnst54/1000000)-o1"
"spoff28=bf1*(cnst54/1000000)-o1"
"spoff29=bf1*(cnst54/1000000)-o1"
"spoff30=0"

aqseq 321

"acqt0=0"
baseopt_echo

1 d11 ze
 d11 pl26:f3
2 d11 do:f3
3 d1
 50u UNBLKGRAD
 d12 pl0:f1
 (p31:sp31 ph1):f1
  d7 gron0
 4u groff
 d12 pl1:f1
 (p41:sp25 ph1)
 p29:gp3
 d16
 DELTA1
 (center (p42:sp26 ph1) (p56:sp39 ph1):f3 )
 DELTA1
 p29:gp3
 d16
 (p41:sp27 ph2):f1

 p16:gp4
 d16 pl3:f3
 (p21 ph3):f3
 DELTA6
 (center (p14:sp3 ph1):f2 (p57:sp40 ph1):f3 )
```

```
DELTA7
(p44:sp30 ph1)
d26 pl3:f3
(p21 ph1):f3
(p44:sp30 ph1)
p16:gp5
d16
 (p44:sp30 ph1)
(p13:sp2 ph4):f2
d0
(center (p44:sp30 ph1) (p14:sp5 ph1):f2 (p56:sp39 ph7):f3 )
d0
4u
(p14:sp3 ph1):f2
DELTA
(p14:sp5 ph1):f2
4u
(p13:sp8 ph1):f2
p16:gp6
d16 pl3:f3
(p44:sp30 ph1)
(p21 ph8):f3
2u
(p56:sp39 ph1):f3
d10
(p14:sp5 ph1):f2
DELTA8
(p44:sp30 ph1)
DELTA2
p16:gp1*EA
d16
(p14:sp3 ph1):f2
d29
(p56:sp39 ph7):f3
d30
2u pl3:f3
(p43:sp28 ph1)
(p21 ph5):f3
p19:gp7
d16
DELTA3
(center (p42:sp26 ph1) (p57:sp40 ph1):f3 )
DELTA3
p19:gp7
d16 pl3:f3
(p21 ph6):f3
(p43:sp29 ph2)
p29:gp8
d16
DELTA4
(center (p42:sp26 ph1) (p56:sp39 ph1):f3 )
DELTA4
p29:gp8
d16
(p43:sp28 ph1)
DELTA5
(p42:sp26 ph1)
4u
p16:gp2
d16 pl26:f3
4u BLKGRAD
go=2 ph31 cpd3:f3
d11 do:f3 mc #0 to 2
  F1PH(calph(ph4, +90), caldel(d0, +in0))
  F2EA(calgrad(EA) & calph(ph6, +180), caldel(d10, +in10) & caldel(d29, +in29) & caldel(d30, -in30) & calph(ph8, +180) & calph(ph31, +180))
TAU
```

```
exit


ph1=0
ph2=1
ph3=0 0 0 0 2 2 2 2
ph4=0 2
ph5=0 0 2 2
ph6=1 1 3 3
ph7=0 0 0 0 2 2 2 2
ph8=0
ph31=0 2 2 0 2 0 0 2


;pl0 : 0W
;pl3 : f3 channel - power level for pulse (default)
;pl26: f3 channel - power level for CPD/BB low power decoupling
;sp2: f2 channel - shaped pulse  90 degree  (Ca on resonance)
;sp3: f2 channel - shaped pulse 180 degree  (Ca on resonance)
;sp5: f2 channel - shaped pulse 180 degree  (C=O off resonance)
;sp8: f2 channel - shaped pulse  90 degree  (Ca on resonance)
;               for time reversed pulse
;sp25: f1 channel - shaped pulse  90 degree (Pc9_4_90.1000)
;sp26: f1 channel - shaped pulse 180 degree (Reburp.1000)
;sp27: f1 channel - shaped pulse  90 degree (Pc9_4_90.1000)
;                for time reversed pulse
;sp28: f1 channel - shaped pulse  90 degree (Eburp2.1000)
;sp29: f1 channel - shaped pulse  90 degree (Eburp2tr.1000)
;                for time reversed pulse
;sp30: f1 channel - shaped pulse 180 degree (Bip720,50,20.1)
;sp31: f1 channel - shaped pulse 180 degree (Reburp.1000)
;sp39: f3 channel - shaped pulse 180 degree (Bip720,50,20.1)
;sp40: f3 channel - shaped pulse 180 degree (Reburp.1000)
;p13: f2 channel -  90 degree shaped pulse
;p14: f2 channel - 180 degree shaped pulse
;p16: homospoil/gradient pulse                      [1 msec]
;p19: gradient pulse 2                              [500 usec]
;p21: f3 channel -  90 degree high power pulse
;p29: gradient pulse 3                              [250 usec]
;p31: f1 channel - shaped pulse 180 degrees for selective inversion recovery
;p41: f1 channel -  90 degree shaped pulse for excitation
;                  Pc9_4_90.1000            (2.2ms at 600.13 MHz)
;p42: f1 channel - 180 degree shaped pulse for refocussing
;                  Reburp.1000              (1.4ms at 600.13 MHz)
;p43: f1 channel -  90 degree shaped pulse for excitation
;                  Eburp2.1000/Eburp2tr.1000 (1.7ms at 600.13 MHz)
;p44: f1 channel - 180 degree shaped pulse for refocussing
;                  Bip720,50,20.1           (200us at 600.13 MHz)
;p56: f3 channel - 180 degree shaped pulse for inversion
;                  Bip720,50,20.1           (500us at 600.13 MHz)
;p57: f3 channel - 180 degree shaped pulse for refocussing
;                  Reburp.1000              (1.6ms at 600.13 MHz)
;d0 : incremented delay (F1 in 3D)                  [3 usec]
;d1 : relaxation delay; 1-5 * T1
;d7 : recovery delay after inversion
;d10: incremented delay (F2 in 3D)                  [3 usec]
;d11: delay for disk I/O                            [30 msec]
;d12: delay for power switching                     [20 usec]
;d16: delay for homospoil/gradient recovery
;d23: 1/(4J(NCa)                                    [12 msec]
;d26: 1/(4J(NH)                                     [2.7 msec]
;d29: incremented delay (F2 in 3D)                  [3 usec]
;d30: decremented delay (F2 in 3D) = d23-p43-4u-p21*4/PI
;cnst21: CO chemical shift (offset, in ppm)
;cnst22: Calpha chemical shift (offset, in ppm)
;cnst41: compensation of chemical shift evolution during p41
;         Pc9_4_90.1000: 0.529
```

```
;cnst43: compensation of chemical shift evolution during p43
;          Eburp2.1000: 0.5
;cnst51: scaling factor for p41 to compensate for transition region
;          Pc9_4_90.1000: 1.172
;cnst52: scaling factor for p42 to compensate for transition region
;          Reburp.1000: 1.426
;cnst53: scaling factor for p43 to compensate for transition region
;          Eburp2.1000: 1.000
;cnst54: H(N) chemical shift (offset, in ppm)
;cnst55: H(N) bandwidth (in ppm)
;o2p: Ca chemical shift (cnst22)
;inf1: 1/SW(Ca) = 2 * DW(Ca)
;inf2: 1/SW(N) = 2 * DW(N)
;in0: 1/(2 * SW(Ca)) =  DW(Ca)
;nd0: 2
;in10: 1/(2 * SW(N)) = DW(N)
;nd10: 2
;in29: = (1 - k2) * in10
;in30: = k2 * in10
;ns: 8 * n
;ds: >= 16
;aq: <= 50 msec
;td1: number of experiments in F1
;td2: number of experiments in F2
;FnMODE: States-TPPI (or TPPI) in F1
;FnMODE: echo-antiecho in F2
;cpd3: decoupling according to sequence defined by cpdprg3: garp4.p62
;pcpd3: f3 channel - 90 degree pulse for decoupling sequence
;cpdprg3: garp4.p62


;for z-only gradients:
;gpz0: 1%
;gpz1: 80%
;gpz2: 8.1%
;gpz3: 11%
;gpz4: 70%
;gpz5: 40%
;gpz6: 75%
;gpz7: 29%
;gpz8: 17%

;use gradient files:
;gpnam1: SMSQ10.100
;gpnam2: SMSQ10.100
;gpnam3: SMSQ10.32
;gpnam4: SMSQ10.100
;gpnam5: SMSQ10.100
;gpnam6: SMSQ10.100
;gpnam7: SMSQ10.50
;gpnam8: SMSQ10.32

                                        ;preprocessor-flags-start
;CALC_SP: for calculation of all bandselective Proton pulses based on cnst54 and cnst55
;            option -DCALC_SP (eda: ZGOPTNS)
                                        ;preprocessor-flags-end



;$Id:$
```

4. Bibliography

(1) Sivashanmugam, A.; Murray, V.; Cui, C.; Zhang, Y.; Wang, J.; Li, Q. Practical protocols for production of very high yields of recombinant proteins using Escherichia coli. *Prot. Sci.* **2009**, *18* (5), 936–948. https://doi.org/10.1002/pro.102.

(2) Marley, J.; Lu, M.; Bracken, C. A method for efficient isotopic labeling of recombinant proteins. *J. Biomol. NMR* **2001**, *20* (1), 71–75. https://doi.org/10.1023/A:1011254402785.

(3) Weisemann, R.; Rüterjans, H.; Bermel, W. 3D triple-resonance NMR techniques for the sequential assignment of NH and ^{15}N resonances in ^{15}N- and ^{13}C-labelled proteins. *J. Biomol. NMR.* **1993**, *3* (1). https://doi.org/10.1007/BF00242479.

(4) Bracken, C.; III, A. G. P.; Cavanagh, J. (H)N(COCA)NH and HN(COCA)NH experiments for ^{1}H-^{15}N backbone assignments in ^{13}C/^{15}N-labeled proteins. *J. Biomol. NMR.* **1997**, *9* (1), 94–100. https://doi.org/10.1023/A:1018679819693.

(5) Bhavesh, N. S.; Panchal, S. C.; Hosur, R. V. An efficient high-throughput resonance assignment procedure for structural genomics and protein folding research by NMR. *Biochemistry.* **2001**, *40* (49), 14727–14735. https://doi.org/10.1021/bi015683p.

(6) Sun, Z.-Y. J.; Frueh, D. P.; Selenko, P.; Hoch, J. C.; Wagner, G. Fast assignment of ^{15}N-HSQC peaks using high-resolution 3D HNcocaNH experiments with non-uniform sampling. *J. Biomol. NMR.* **2005**, *33* (1), 43–50. https://doi.org/10.1007/s10858-005-1284-4.

(7) Slad, S.; Bermel, W.; Kümmerle, R.; Mathieu, D.; Luy, B. Band-selective universal 90° and 180° rotation pulses covering the aliphatic carbon chemical shift range for triple resonance experiments on 1.2 GHz Spectrometers. *J. Biomol. NMR.* **2022**, *76* (5–6), 185–195. https://doi.org/10.1007/s10858-022-00404-1.

(8) Bermel, W.; Bertini, I.; Duma, L.; Felli, I. C.; Emsley, L.; Pierattelli, R.; Vasos, P. R. Complete assignment of heteronuclear protein resonances by protonless NMR spectroscopy. *Angew. Chem. Int. Ed.* **2005**, *44* (20), 3089–3092. https://doi.org/10.1002/anie.200461794.

(9) Pontoriero, L.; Schiavina, M.; Murrali, M. G.; Pierattelli, R.; Felli, I. C. Monitoring the interaction of α-synuclein with calcium ions through exclusively heteronuclear nuclear magnetic resonance experiments. *Angew. Chem. Int. Ed.* **2020**, *59* (42), 18537–18545. https://doi.org/10.1002/anie.202008079.

(10) Bermel, W.; Bertini, I.; Felli, I. C.; Kümmerle, R.; Pierattelli, R. Novel ^{13}C direct detection experiments, including extension to the third dimension, to perform the complete assignment of proteins. *J. Magn. Reson.* **2006**, *178* (1), 56–64. https://doi.org/10.1016/j.jmr.2005.08.011.

(11) Emsley, L.; Bodenhausen, G. Optimization of shaped selective pulses for NMR using a quaternion description of their overall propagators. *J. Magn. Reson.* **1992**, *97* (1), 135–148. https://doi.org/10.1016/0022-2364(92)90242-Y.

(12) Felli, I. C.; Pierattelli, R. Spin-state-selective methods in solution- and solid-state biomolecular ^{13}C NMR. *Prog. Nucl. Magn. Reson. Spectrosc.* **2015**, *84–85*, 1–13. https://doi.org/10.1016/j.pnmrs.2014.10.001.

(13) Kadkhodaie, M.; Rivas, O.; Tan, M.; Mohebbi, A.; Shaka, A. J. Broadband homonuclear cross polarization using flip-flop spectroscopy. *J. Magn. Reson.* **1991**, *91* (2), 437–443. https://doi.org/10.1016/0022-2364(91)90210-K.

(14) Schanda, P.; Van Melckebeke, H.; Brutscher, B. Speeding up three-dimensional protein NMR experiments to a few minutes. *J. Am. Chem. Soc.* **2006**, *128* (28), 9042–9043. https://doi.org/10.1021/ja062025p.

(15) Grzesiek, S.; Bax, A. Improved 3D triple-resonance NMR techniques applied to a 31 KDa protein. *J. Magn. Reson.* **1992**, *96* (2), 432–440. https://doi.org/10.1016/0022-2364(92)90099-S.

(16) Schleucher, J.; Sattler, M.; Griesinger, C. Coherence selection by gradients without signal attenuation: application to the three-dimensional HNCO experiment. *Angew. Chem. Int. Ed.* **1993**, *32* (10), 1489–1491. https://doi.org/10.1002/anie.199314891.

(17) Kay, L. E.; Xu, G. Y.; Yamazaki, T. Enhanced-sensitivity triple-resonance spectroscopy with minimal H_2O saturation. *J. Magn. Reson.* **1994**, *109* (1), 129–133. https://doi.org/10.1006/jmra.1994.1145.

(18) Clubb, R. T.; Thanabal, V.; Wagner, G. A constant-time three-dimensional triple-resonance pulse scheme to correlate intraresidue $^1H^N$, ^{15}N, and $^{13}C'$ chemical shifts in ^{15}N- ^{13}C-labelled proteins. *J. Magn. Reson.* **1992**, *97* (1), 213–217. https://doi.org/10.1016/0022-2364(92)90252-3.

(19) Grzesiek, S.; Bax, A. An efficient experiment for sequential backbone assignment of medium-sized isotopically enriched proteins. *J. Magn. Reson.* **1992**, *99* (1), 201–207. https://doi.org/10.1016/0022-2364(92)90169-8.

(20) Grzesiek, S.; Bax, A. Amino acid type determination in the sequential assignment procedure of uniformly $^{13}C/^{15}N$-enriched proteins. *J. Biomol. NMR.* **1993**, *3* (2). https://doi.org/10.1007/BF00178261.

(21) Muhandiram, D. R.; Kay, L. E. Gradient-enhanced triple-resonance three-dimensional NMR experiments with improved sensitivity. *J. Magn. Reson.* **1994**, *103* (3), 203–216. https://doi.org/10.1006/jmrb.1994.1032.

(22) Bermel, W.; Bertini, I.; Csizmok, V.; Felli, I. C.; Pierattelli, R.; Tompa, P. H-start for exclusively heteronuclear NMR spectroscopy: the case of intrinsically disordered proteins. *J. Magn. Reson.* **2009**, *198* (2), 275–281. https://doi.org/10.1016/j.jmr.2009.02.012.

(23) Banci, L.; Benedetto, M.; Bertini, I.; Del Conte, R.; Piccioli, M.; Viezzoli, M. S. Solution structure of reduced monomeric Q133M2 copper, zinc superoxide dismutase (SOD). Why is SOD a dimeric enzyme?. *Biochemistry.* **1998**, *37* (34), 11780–11791. https://doi.org/10.1021/bi9803473.

(24) Christou, N. E.; Brutscher, B. BEST and SOFAST experiments for resonance assignment of histidine and tyrosine side chains in $^{13}C/^{15}N$ labeled proteins. *J. Biomol. NMR*. **2018**, *72* (3–4), 115–124. https://doi.org/10.1007/s10858-018-0216-z.

(25) Pervushin, K.; Riek, R.; Wider, G.; Wüthrich, K. Transverse relaxation-optimized spectroscopy (TROSY) for NMR studies of aromatic spin systems in ^{13}C-labeled proteins. *J. Am. Chem. Soc.* **1998**, *120* (25), 6394–6400. https://doi.org/10.1021/ja980742g.

(26) Bartels, C.; Xia, T.; Billeter, M.; Güntert, P.; Wüthrich, K. The program XEASY for computer-supported NMR spectral analysis of biological macromolecules. *J. Biomol. NMR.* **1995**, *6* (1), 1–10. https://doi.org/10.1007/BF00417486.

(27) García de la Torre, J.; Huertas, M. L.; Carrasco, B. HYDRONMR: prediction of NMR relaxation of globular proteins from atomic-level structures and hydrodynamic calculations. *J. Magn. Reson.* **2000**, *147* (1), 138–146. https://doi.org/10.1006/jmre.2000.2170.

(28) De Guzman, R. N.; Liu, H. Y.; Martinez-Yamout, M.; Dyson, H. J.; Wright, P. E. Solution structure of the TAZ2 (CH3) domain of the transcriptional adaptor protein CBP. *J. Mol. Biol.* **2000**, *303* (2), 243–253. https://doi.org/10.1006/jmbi.2000.4141.

(29) Teixeira, J. M. C.; Liu, Z. H.; Namini, A.; Li, J.; Vernon, R. M.; Krzeminski, M.; Shamandy, A. A.; Zhang, O.; Haghighatlari, M.; Yu, L.; Head-Gordon, T.; Forman-Kay, J. D. IDPConformerGenerator: a flexible software suite for sampling the conformational space of disordered protein states. *J. Phys. Chem. A.* **2022**, *126* (35), 5985–6003. https://doi.org/10.1021/acs.jpca.2c03726.

(30) Wang, G.; Dunbrack, R. L. PISCES: a protein sequence culling server. *Bioinformatics.* **2003**, *19* (12), 1589–1591. https://doi.org/10.1093/bioinformatics/btg224.

(31) Liu, Z. H.; Teixeira, J. M. C.; Zhang, O.; Tsangaris, T. E.; Li, J.; Gradinaru, C. C.; Head-Gordon, T.; Forman-Kay, J. D. Local Disordered Region Sampling (LDRS) for ensemble modeling of proteins with experimentally undetermined or low confidence prediction segments. *Bioinformatics.* **2023**, *39* (12). https://doi.org/10.1093/bioinformatics/btad739.

(32) Abramson, J.; Adler, J.; Dunger, J.; Evans, R.; Green, T.; Pritzel, A.; Ronneberger, O.; Willmore, L.; Ballard, A. J.; Bambrick, J.; Bodenstein, S. W.; Evans, D. A.; Hung, C.-C.; O'Neill, M.; Reiman, D.; Tunyasuvunakool, K.; Wu, Z.; Žemgulytė, A.; Arvaniti, E.; Beattie, C.; Bertolli, O.; Bridgland, A.; Cherepanov, A.; Congreve, M.; Cowen-Rivers, A. I.; Cowie, A.; Figurnov, M.; Fuchs, F. B.; Gladman, H.; Jain, R.; Khan, Y. A.; Low, C. M. R.; Perlin, K.; Potapenko, A.; Savy, P.; Singh, S.; Stecula, A.; Thillaisundaram, A.; Tong, C.; Yakneen, S.; Zhong, E. D.; Zielinski, M.; Žídek, A.; Bapst, V.; Kohli, P.; Jaderberg, M.; Hassabis, D.; Jumper, J. M. Accurate structure prediction of biomolecular interactions with AlphaFold 3. *Nature.* **2024**, *630* (8016), 493–500. https://doi.org/10.1038/s41586-024-07487-w.

(33) Hwang, T. L.; Shaka, A. J. Water suppression that works. Excitation sculpting using arbitrary wave-forms and pulsed-field gradients. *J. Magn. Reson.* **1995**, *112* (2), 275–279. https://doi.org/10.1006/jmra.1995.1047.

(34) Schleucher, J.; Schwendinger, M.; Sattler, M.; Schmidt, P.; Schedletzky, O.; Glaser, S. J.; Sorensen, O. W.; Griesinger, C. A General enhancement scheme in heteronuclear multidimensional NMR employing pulsed field gradients. *J. Biomol. NMR.* **1994**, *4* (2). https://doi.org/10.1007/BF00175254.

(35) Brutscher, B.; Solyom, Z. Chapter 1. Polarization-enhanced fast-pulsing techniques. In *Fast NMR data acquisition: beyond the Fourier transfrom*; Mobli, M., Hoch, J. C., Eds.; Royal society of chemistry, 2017; pp 1–32. https://doi.org/10.1039/9781782628361-00001.

(36) Tamiola, K.; Mulder, F. A. A. Using NMR chemical shifts to calculate the propensity for structural order and disorder in proteins. *Biochem. Soc. Trans.* **2012**, *40* (5), 1014–1020. https://doi.org/10.1042/BST20120171.

(37) Madeira, F.; Madhusoodanan, N.; Lee, J.; Eusebi, A.; Niewielska, A.; Tivey, A. R. N.; Lopez, R.; Butcher, S. The EMBL-EBI job dispatcher sequence analysis tools framework in 2024. *Nucleic Acids Res.* **2024**, *52* (W1), W521–W525. https://doi.org/10.1093/nar/gkae241.
